# Quality of life, health perception, learning motivation and behavior of adolescents in an educational institution ^*^

**DOI:** 10.1590/1518-8345.6919.4338

**Published:** 2024-10-25

**Authors:** Graziela Nunes Alfenas Fernandes, Stela Maris Aguiar Lemos

**Affiliations:** ^1^ Universidade Federal de Minas Gerais, Faculdade de Medicina, Belo Horizonte, MG, Brazil.; ^2^ Scholarship holder at the Coordenação de Aperfeiçoamento de Pessoal de Nível Superior (CAPES), Brazil.

**Keywords:** Adolescent, Health, Quality of Life, Behaviour, Remote Teaching, Perception

## Abstract

**Objective:**

to analyze sociodemographic variables, quality of life, self-perceived health, learning motivation and behavior of adolescents in 2018 and 2021.

**Method:**

observational cross-sectional study with 124 adolescents in 2018, and 68 in 2021. A Form for sociodemographic variables, Pediatric Quality of Life Inventory, Self-Perceived Health instrument, Learning Motivation Scale and Strengths and Difficulties Questionnaire were used to collect data. Data collection was carried out using forms on Google Forms. For the analysis, descriptive statistics and logistic regression were used.

**Results:**

the majority of participants belonged to class A. In the comparison between 2018 and 2021, there was a worsening in the assessment of self-perceived health. In the assessment of QoL by parents, there was a statistically significant difference between the scores of the social and psychosocial dimensions. Among adolescents, there were differences between QoL scores in the physical and psychosocial dimensions.

**Conclusion:**

the adolescent with a better mental health assessment had a greater chance of having a better quality of life in the periods investigated (OR=5.35 and OR=5.51). Younger students showed greater motivation to learn, increasing the chance of improving their quality of life by up to 9.75 and 5.02 times in the two periods, respectively.

## Highlights

**(1)** The school environment is a space favorable to promoting physical, mental and social health.

**(2)** Awareness about healthy lifestyle habits should be carried out during adolescence.

**(3)** Aspects related to health can influence behavior and learning.

**(4)** Quality of life (QoL) and its dimensions are related to the motivation to learn.

**(5)** Adolescent mental health is related to self-perception of QoL.

## Introduction

 A major Public Health crisis brought significant global changes between 2019 and 2021 in terms of the way people live and coexist, creating the immediate need for adaptations of all kinds. The pandemic caused by Severe Acute Respiratory Syndrome coronavirus 2 (SARS-CoV-2) changed the way of thinking and practicing education in Brazil and around the world, affecting more than 90% of students across the planet and educational processes in more than 180 countries. In Brazil, social distancing measures to mitigate the virus resulted in the suspension of in-person school activities and government decrees authorized Emergency Remote Education (ERE), suddenly implementing school activities through information and communication technologies ^(^ ^1^ ^-^ ^3^ ^)^ . 

 The individual, during childhood and adolescence, finds school to be an important dimension of their quality of life, as in this space, in addition to acquiring curricular knowledge, they develop fundamental values for their integral formation as human being and citizen. In this sense, the school environment is conducive to carrying out effective and comprehensive health promotion and education programs ^(^ ^4^ ^)^ , as, through intersectorality ^(^ ^5^ ^-^ ^6^ ^)^ , it cooperates to build knowledge about human nature and its development, improving skills and creating capabilities for self-care, environmental care and general well-being ^(^ ^7^ ^-^ ^9^ ^)^ and brings together cognitive, physical, emotional, family and socioeconomic demands of students towards comprehensive health. 

 Thus, highlighting the school as a place of care, stimulation and growth in the different areas of life, the new format of knowledge construction brought reflections on fundamental characteristics of this didactic-pedagogical process, involving technical, teaching and student teams around teaching and learning ^(^ ^10^ ^)^ . It is known that human motivation to perform tasks follows internal stimuli and contextual aspects. During adolescence, a period characterized by transitions, motivation is essential to enable young people to achieve their goals and experience healthy development, including psychological needs, self-discipline and academic encouragement ^(^ ^11^ ^)^ . In the context of learning, motivation can be mainly focused on individual improvement or mostly aimed at performance in carrying out academic activities ^(^ ^12^ ^)^ . Combined with the motivation to learn, factors related to mental health ^(^ ^13^ ^)^ , behavior, family aspects ^(^ ^14^ ^)^ and socioeconomic and demographic issues ^(^ ^15^ ^)^ can influence the educational process of adolescents ^(^ ^2^ ^)^ . 

 Studies carried out in Brazil ^(^ ^16^ ^-^ ^18^ ^)^ and in countries such as China and Germany ^(^ ^19^ ^-^ ^21^ ^)^ showed worsening of mental health, self-esteem and psychosocial well-being of adolescents during the pandemic. The negative effects impacted development and performance in different areas of life. It is also worth highlighting the consequences of these factors for the general quality of life and its physical, emotional, academic and social dimensions in this population, accentuated by the implementation of new pedagogical and didactic strategies inherent to the emergency education model. 

Thus, thinking about basic education and rescuing teaching and learning as interaction processes that permeate attachment, biopsychosocial development and school, the main objective of this study was to analyze sociodemographic variables, quality of life, self-perceived health, learning motivation and behavior of adolescents from a private institution before the COVID-19 pandemic, in 2018 and 2021.

## Method

### Study design and sample definition

This is a quantitative observational cross-sectional study with a sample stratified by gender, age and school grade. The sample consisted of 124 adolescents enrolled in Elementary Education II at a privately funded Brazilian school before the COVID-19 pandemic in 2018, and 68 students during the COVID-19 pandemic in 2021, where approximately 244 students were enrolled in the educational segment researched.

The sample calculation considered 5% sampling error, 95% confidence interval and 15% prevalence considering quality of life as the outcome of interest, estimating a sample of 114 adolescents selected by random sampling stratified by gender and school grade. A test was used to estimate a proportion in the Minitab 14 Release software.

For learning motivation, a sample size of 124 individuals obtained 80% statistical power in estimating low motivation to learn, considering 22.5% as the parameter in the population. The precision obtained from this sample size and statistical power is 10% and the significance level was 5%. A test was used to estimate a proportion in the Minitab 17 software.

### Selection criteria

**Figure 1:**
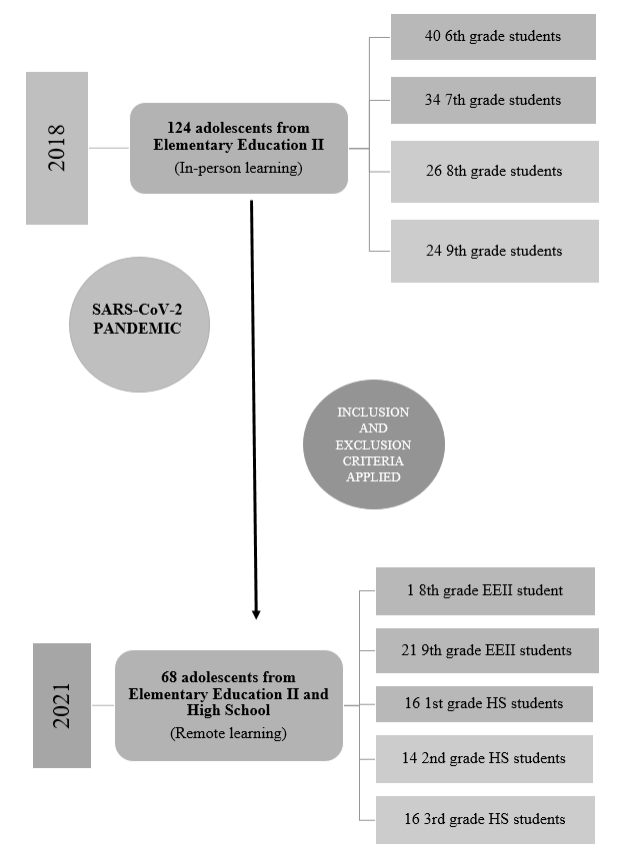
- Flowchart of research participants 2018-2021

^*^ EEII = Elementary Education II; ^†^ HS = High School

 As inclusion criteria, in 2018, adolescents enrolled in the Elementary Education II segment at the researched institution were considered, aged between 11 and 14 years old and students in the 6 ^th^ to 9 ^th^ school grades, with 124 adolescents included. Failure to complete the research instruments was used as an exclusion criterion. In 2021, all 124 participants included in 2018 were invited to participate in the second stage of the study, 56 refused for reasons such as changing schools, lack of time to complete the research instruments and changing telephone numbers, and 68 agreed with the new data collection ( Figure 1 ). 

### Ethical aspects

 This research was approved by the Research Ethics Committee of the *Universidade Federal de Minas Gerais* (UFMG) under Certificate of Presentation for Ethical Consideration (CAAE, acronym in Portuguese) number 80162417.1.0000.5149. The project was approved under opinion number 2.422.795 in December 2017 and the project amendment was approved under opinion number 4.446.496 in December 2020. In 2018, the parents or guardians and the participating adolescents signed the Free and Informed Consent Term (TCLE, acronym in Portuguese) and the Free and Informed Assent Term (TALE, acronym in Portuguese), respectively. In 2021, due to emergency remote learning, participants accepted the TCLE and TALE through electronic forms. 

### Study variables

 For this study, participants’ characterization information - gender, age, school grade and economic classification (CCEB) -, self-perceived health and strengths and difficulties (SDQ – Por) were considered as explanatory variables, and quality of life, self-report and report of parents in the physical, psychosocial and general quality of life dimensions (PedsQL ^TM^ ) and motivation to learn, in its three domains (EMAPRE, acronym in Portuguese), as response variables. 

### Instruments used to collect information

In this study, five instruments were used: a questionnaire containing the participants’ sociodemographic variables, the Pediatric Quality of Life Inventory – PedsQL™ 4.0, the questionnaire on Self-Perceived Health, the Learning Motivation Scale – EMAPRE and The Strengths and Difficulties Questionnaire – SDQ.

 Form to obtain sociodemographic variables (gender, age and school grade and economic classification). To classify the economic class, the *Critério de Classificação Econômica Brasil* - CCEB ^(^ ^22^ ^)^ was adopted. To this end, the parents and/or guardians of the adolescents were asked to fill out the items referring to the purchasing power and level of education of the head of the family, only in 2018, in order to later group the participants into classes A to E. 

 Pediatric Quality of Life Inventory - PedsQL™ 4.0. This instrument was standardized and validated for Brazilian Portuguese ^(^ ^23^ ^-^ ^24^ ^)^ , and allows the assessment of quality of life (QoL) in four domains: physical (physical dimension), emotional, social and school (psychosocial dimension). For its use, permission was requested from the authors. In addition to the version intended for parents to complete, this questionnaire also has a version intended for adolescents, for self-reporting of quality of life, and both were used in the research. To obtain the score, the items are inverted and linearly transformed into a scale from 0 to 100: 0 - 100; 1 - 75; 2 - 50; 3 - 25; 4 - 0. Then, the scores for each item are added and divided by the number of scores answered, presenting the average for each item in the domain. To obtain data on the Psychosocial Dimension, the scores on the Emotional, Social and School scales are added together. For the Physical Dimension, the answers given on the physical scale are used. For the overall score, the calculation is performed by dividing the sum of all items by the number of items answered on all scales. 

Questionnaire on Self-Perceived Health. This questionnaire was prepared by the researchers with the following questions: “How do you currently evaluate/consider your health?” and “How would you rate your health?”. Answers to the first question are evaluated using a Likert scale with the options: very poor, poor, fair, good and excellent. For the second question, a numerical scale from 0 to 10 was used to assign the answer, with zero being considered very poor and 10 being excellent.

 Learning Motivation Scale – EMAPRE ^(^ ^12^ ^)^ . This instrument allows students to evaluate their motivation to study and carry out academic activities. The scale consists of 28 questions distributed across three domains: Learning Goal, Performance-approach Goal and Performance-avoidance Goal. The Learning Goal is one in which the student seeks challenges and uses them as a resource for their own learning and intellectual development. The Performance-approach Goal highlights the student’s concern mainly with surpassing others by demonstrating their own ability. And the Performance-avoidance Goal refers to the condition in which the student avoids situations where failure may occur in carrying out the proposed school tasks. Thus, all questions relate to motivation, attitude and objectives regarding learning. EMAPRE is a Likert-type scale, with response options “agree”, “don’t know” and “disagree”. This instrument was developed with High School students, and was subjected to factor analysis in which the three factors explained 40.56% of the total variance in the analysis of the main components, and the three domains presented Cronbach’s Alpha α=0.795, α=0.798 and α=0.801, respectively, in the analysis of internal consistency, demonstrating the suitability of the instrument for the sample of adolescents proposed in the study ^(^ ^25^ ^)^ . 

 Strengths and Difficulties Questionnaire (SDQ) ^(^ ^26^ ^)^ . This instrument was constructed in 1997 by Goodman and validated in Brazil by Fleitlich, Cortázar and Goodman in 2000. The SDQ is a questionnaire that allows tracking child and adolescent mental health problems. The questionnaire consists of 25 items, distributed across five scales, namely: emotional symptoms (five items); behavior problems (five items); hyperactivity/inattention (five items); relationship problems (five items) and prosocial behavior (five items). Of these 25 items, 10 are about capabilities, 14 about difficulties and one of them is considered neutral. Each of the items can be answered as “false”, “more or less true” or “true”. The score for each of the scales is obtained by adding the scores of the 5 items, generating a score that varies from 0 to 10. Scores on the hyperactivity, emotional symptoms, conduct problems and peer relationship problems scales are added together to generate a total difficulties score, which varies between 0 and 40. A total score greater than or equal to 20 is considered, according to the author, as altered, between 16 and 19, borderline, and less than or equal to 15, normal, which shows the absence of psychological difficulties. The prosocial behavior scale score is not incorporated into the total difficulties score, as the absence of prosocial behaviors is conceptually different from the presence of psychological difficulties. The interpretation of the score on this scale differs because higher scores mean a greater frequency of prosocial behaviors, unlike the total score, where higher values represent more difficulties. The SDQ can be answered by parents, teachers and children themselves, over 11 years of age. In this survey, the adolescents themselves answered the questions. 

### Data collection

In 2018, data collection was carried out between June and August using forms created in Google Forms and applied during the adolescents’ timetable and school environment. In 2021, data collection was carried out between April and June with forms distributed via email and WhatsApp, due to emergency remote learning. The sequence of application of the instruments for the students was the Participant Characterization Form, the Self-Perceived Health Questionnaire, the Pediatric Quality of Life Inventory, the Strengths and Difficulties Questionnaire (SDQ) and the Learning Motivation Scale (EMAPRE).

### Data processing and analysis

For data analysis, the information was carefully entered into the research database and subjected to triple typing, in order to minimize the risk of errors. Descriptive, bivariate and multivariate analyzes were performed. The descriptive analysis considered the absolute and relative frequency distribution of the categorical ones (gender, age, school grade, economic classification, self-perceived health, SDQ, quality of life, learning motivation) and the numerical synthesis of the continuous ones (health score). Comparative analyzes between the two periods were carried out considering the response and explanatory variables and, to this end, the Wilcoxon test was used for continuous variables, as they all had an asymmetrical distribution, and the McNemar test for categorical variables.

For bivariate analysis, Pearson’s Chi-square or Fisher’s Exact tests were used for categorical variables, considering a significance level of 5%. The variables with an association at the 20% level of significance were initially considered for the multivariate analysis. To select variables in the models, the manual backward method was adopted, considering at each step of the analysis the variable with the highest p-value for removal from the model. In the final model, variables with a significant association at the 5% level and the age variable were maintained, which remained as an adjustment variable. The magnitude of the associations was evaluated by the Odds Ratio (OR) and their respective 95% confidence intervals. P values lower than 5% were considered statistically significant and, for data analysis, the statistical program Statistical Package for the Social Sciences (SPSS) was used. The adequacy of the models was assessed using the Hosmer and Lemeshow test.

## Results

**Figure 2:**
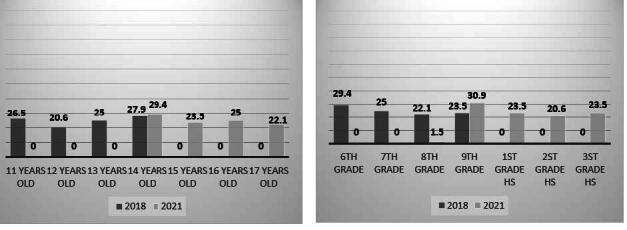
- Distribution of sociodemographic variables (gender, age and school grade and economic classification) of the adolescents (n = 68). Belo Horizonte, MG, Brazil, 2018 and 2021

^*^ HS = High School

 Of the 124 participants, the majority of participants were female (57.4%) and their age varied uniformly between 11 and 14 years in the first stage and between 14 and 17 years in the second. The majority of adolescents (29.4%) were in the 6th grade and 30.9% in the 9th grade, in 2018 and 2021, respectively. Regarding economic classification, 58.8% of adolescents were in class A ( Figure 2 ). 

As for self-perceived health, in 2018, 91.2% considered that they had good or excellent health, and in 2021 this value was reduced to 80.9%, but without a statistically significant difference (p=0.118). The average of responses about the value attributed to their health (zero to 10) remained stable in both periods at 8.82 (Standard deviation (SD) =1.12 in 2018 and SD=1.23 in 2021), as well as the median (9.00).

Regarding strengths and difficulties, both the total score on the SDQ scale and the pro-social behavior score had a large majority of “Normal” responses, and there was no significant difference in the percentage of responses between the categories at the two moments of the study. Regarding the assessment of QoL, the analyzes covered all its dimensions: physical, psychosocial (emotional, social and school) and general. In QoL assessed by parents, there was a statistically significant difference between the scores of the social (p=0.003) and psychosocial (p=0.039) dimensions, both with higher scores in 2021. Among adolescents, there were differences between QoL scores in the physical, social, school (p=0.006), emotional (p=0.008) and psychosocial (p=0.013) dimensions. In the categorical variables, the difference was significant for the physical dimension score (p<0.001).

In the inferential analysis, when considering parents’ responses, in 2018 there was a significant association between the physical dimension and the school grade (p=0.027) and the total SDQ classification (p=0.056). In 2021, this dimension was associated with self-perceived health (p=0.031) and the Performance-approach Goal (p=0.005), and bordering on gender (p=0.058). In the psychosocial dimension assessed by parents, there was a significant association in 2018 with self-perceived health (p=0.035) and a borderline association with the total SDQ classification (difficulties) (p=0.054), and in 2021 there was no significant association at the level of 5% between this domain and the tested variables. Regarding general QoL assessed by parents, in 2018 it was associated with self-perceived health (p=0.021) and the total SDQ classification (p=0.027), and in 2021, with the same variables, self-perceived health (p =0.043) and total SDQ classification (p=0.034).

 When considering the adolescents’ responses, in 2018 there was a significant association between the physical dimension and the total SDQ classification (p=0.003), and in 2021 with self-perceived health (p=0.001). In the psychosocial dimension assessed by adolescents, there was a significant association in 2018 with age (p=0.036), school grade (p=0.021) and Learning Goal (p=0.015), with the total SDQ classification (p) and borderline with Performance-avoidance Goal (p=0.054). In 2021, the psychosocial domain was associated with gender (p=0.048), bordering on self-perceived health (p=0.054) and with the total SDQ classification (p=0.009). Regarding the general QoL assessed by adolescents, in 2018 it was associated with age (p=0.045), school grade (p=0.017), Learning Goal (p=0.028) and total SDQ classification (p). In 2021, the general QoL score was associated with self-perceived health (p=0.026), Performance-avoidance Goal (p=0.002) and the total SDQ (p=0.012) (Tables 1 and 2 ). 

 For the multivariate analysis, four models were constructed. In the first model, QoL was assessed based on parents’ perception in 2018 and the variables school grade and total SDQ, with the latter being associated with QoL in the final model. Thus, the normal SDQ result increased the chances of the adolescent having a good/excellent quality of life by 5.37 times in the parents’ perception. In the second model, QoL was evaluated through the perception of adolescents in 2018 and the variables school grade, Learning Goal and Performance-avoidance Goal. In the final model, QoL was associated with the school grade, and being enrolled in the 6 ^th^ and 7 ^th^ grade increased the chances of having a better QoL by 4.5 and 9.7 times, respectively, when compared to students in the 9th grade. In the third model, QoL was assessed according to parents’ perception in 2021. The variables self-perceived health and total SDQ were selected, the latter being associated with QoL in the final model (adjusted for age). The normal SDQ result increased the chances of the adolescent having a good/excellent quality of life by 5.51 times in their parents’ perception. In the fourth model, QoL was evaluated according to the perception of adolescents in 2021, with the variables gender, CCEB, self-perceived health, Performance-avoidance Goal and total SDQ being selected for the initial model. These last two variables remained associated with QoL in the final model, so that having a better result on the performance-avoidance scale (low learning avoidance) increased the chances of having better QoL by 5.02 times, and the normal SDQ result increased by 11 .05 times the chances of the adolescent having better QoL ( Table 3 ). 

**Table 1:** - Bivariate analysis of the association between PedsQL ^*^ variables (parents and adolescents) and selected variables (n = 68). Belo Horizonte, MG, Brazil, 2018

**Characteristics**	**Parents** **Dimensions**						**Adolescents** **Dimensions**					
	**Physical**		**Psychosocial**		**General quality of life**		**Physical**		**Psychosocial**		**General quality of life**	
	high	low	high	low	high	low	high	low	high	low	high	low
	n(%) ^†^	n(%) ^†^	n(%) ^†^	n(%) ^†^	n(%) ^†^	n(%) ^†^	n(%) ^†^	n(%) ^†^	n(%) ^†^	n(%) ^†^	n(%) ^†^	n(%) ^†^
**Gender**												
Female	19 (55.9)	20 (58.8)	21(52.5)	18 (64.3)	19 (51.4)	20 (64.5)	24 (66.7)	15 (46.9)	18 (54.5)	21 (60.0)	17 (53.1)	22 (61.1)
Male	15 (44.1)	14 (41.2)	19 (47.5)	10 (35.7)	18 (48.6)	11 (35.5)	12 (33.3)	17 (53.1)	15 (45.5)	14 (40.0)	15 (46.9)	14 (38.9)
p – Valu ^e‡^	0.806 ^§^		0.333 ^§^		0.274 ^§^		0.100 ^§^		0.649 ^§^		0.506 ^§^	
**Age (years old)**												
11	7 (20.6)	11 (32.4)	10 (25.0)	8 (28.6)	8 (21.6)	10 (32.3)	11 (30.6)	7 (21.9)	7 (21.2)	11 (31.4)	7 (21.9)	11 (30.6)
12	6 (17.6)	8 (23.5)	8 (20.0)	6 (21.4)	8 (21.6)	6 (19.4)	6 (16.7)	8 (25.0)	5 (15.2)	9 (25.7)	5 (15.6)	9 (25.0)
13	8 (23.5)	9 (26.5)	9 (22.5)	8 (28.6)	9 (24.3)	8 (25.8)	7 (19.4)	10 (31.3)	7 (21.2)	10 (28.6)	6 (18.8)	11 (30.6)
14	13 (38.2)	6 (17.6)	13 (32.5)	6 (21.4)	12 (32.5)	7 (22.6)	12 (33.3)	7 (21.9)	14 (42.4)	5 (14.3)	14 (43.8)	5 (13.9)
p – Value ^‡^	0.077 ^||^		0.494 ^||^		0.309 ^||^		0.932 ^||^		**0.036** ^||^		**0.045** ^||^	
**School grade**												
6 ^th^	7 (20.6)	13 (38.2)	10 (25.0)	10 (35.7)	8 (21.6)	12 (38.7)	12 (33.3)	8 (25.0)	8 (24.2)	12 (34.3)	8 (25.0)	12 (33.3)
7 ^th^	7 (20.6)	10 (29.4)	10 (25.0)	7 (25.0)	9 (24.3)	8 (25.8)	5 (13.9)	12 (37.4)	5 (15.2)	12 (34.3)	4 (12.5)	13 (36.1)
8 ^th^	9 (26.5)	6 (17.6)	10 (25.0)	5 (17.9)	11 (29.7)	4 (12.9)	9 (25.0)	6 (18.8)	8 (24.2)	7 (20.0)	8 (25.0)	7 (19.4)
9 ^th^	11 (32.4)	5 (14.7)	10 (25.0)	6 (21.4)	9 (24.3)	7 (22.6)	10 (27.8)	6 (18.8)	12 (36.4)	4 (11.4)	12 (37.5)	4 (11.1)
p – Value ^‡^	**0.027** ^||^		0.377 ^||^		0.181 ^||^		0.567 ^||^		**0.021** ^||^		**0.017** ^||^	
**CCEB** ^¶^												
A1 ^**^	21 (61.8)	19 (55.9)	23 (57.5)	17 (60.7)	21 (56.8)	19 (61.3)	22 (61.1)	18 (56.3)	21 (63.6)	19 (54.3)	20 (62.5)	20 (55.6)
B1/B2 ^††^	13 (38.2)	15 (44.1)	17 (42.5)	11 (39.3)	16 (43.2)	12 (38.7)	14 (38.9)	14 (43.8)	12 (36.4)	16 (45.7)	12 (37.5)	16 (44.4)
p – Value ^‡^	0.622		0.791 ^§^		0.705 ^§^		0.684 ^§^		0.434 ^§^		0.561 ^§^	
**Self-perceived health**												
Poor/Average	5 (14.7)	1 (2.9)	6 (15.0)	0 (0.0)	6 (16.2)	0 (0.0)	4 (11.1)	2 (6.3)	4 (12.1)	2 (5.7)	4 (12.5)	2 (5.6)
Good/Excellent	29 (85.3)	33 (97.1)	34 (85.0)	28 (100.0)	31 (83.8)	31 (100.0)	32 (88.9)	30 (93.7)	29 (87.9)	33 (94.3)	28 (87.5)	34 (94.4)
p – Value ^‡^	0.099 ^‡‡^		**0.035** ^‡‡^		**0.021** ^‡‡^		0.395 ^‡‡^		0.308 ^‡‡^		0.281 ^‡‡^	
**Learning Goal**												
< 31	18 (52.9)	17 (50.0)	21 (52.5)	14 (50.0)	20 (54.1)	15 (48.4)	20 (55.6)	15 (46.9)	22 (66.7)	13 (37.1)	21 (65.6)	14 (38.9)
≥ 31	16 (47.1)	17 (50.0)	19 (47.5)	14 (50.0)	17 (45.9)	16 (51.6)	16 (44.4)	17 (53.1)	11 (33.3)	22 (66.9)	11 (34.4)	22 (61.1)
p – Value ^‡^	0.808 ^§^		0.839 ^§^		0.641 ^§^		0.475 ^§^		**0.015** ^§^		**0.028** ^§^	
**Performance-approach Goal**												
< 15	18 (52.9)	15 (44.1)	20 (50.0)	13 (46.4)	19 (51.4)	14 (45.2)	17 (47.2)	16 (50.0)	15 (45.5)	18 (51.4)	14 (43.7)	19 (52.8)
≥ 15	16 (47.1)	19 (55.9)	20 (50.0)	15 (53.6)	18 (48.6)	17 (54.8)	19 (52.8)	16 (50.0)	18 (54.5)	17 (48.6)	18 (56.3)	17 (47.2)
p – Value ^‡^	0.467 ^§^		0.772 ^§^		0.611 ^§^		0.819 ^§^		0.622 ^§^		0.457 ^§^	
**Performance-avoidance Goal**												
≤ 8	19 (55.9)	18 (52.9)	19 (47.5)	18 (64.3)	19 (51.4)	18 (58.1)	19 (52.8)	18 (56.3)	14 (42.4)	23 (65.7)	14 (43.7)	23 (63.9)
> 8	15 (44.1)	16 (47.1)	21 (52.5)	10 (35.7)	18 (48.6)	13 (41.9)	17 (47.2)	14 (43.8)	19 (57.6)	12 (34.3)	18 (56.3)	13 (36.1)
p – Value ^‡^	0.808 ^§^		0.171 ^§^		0.580 ^§^		0.774 ^§^		**0.054** ^§^		0.096 ^§^	
**Prosocial Behavior** **SDQ** ^§§^												
Normal	33 (97.1)	32 (94.1)	39 (97.5)	26 (92.9)	36 (97.3)	29 (93.5)	34 (94.4)	31 (96.9)	31 (93.9)	34 (97.1)	30 (93.8)	35 (97.2)
Altered ^||^	1 (2.9)	2 (5.9)	1 (2.5)	2 (7.1)	1 (2.7)	2 (6.5)	2 (5.6)	1 (3.1)	2 (6.1)	1 (2.9)	2 (6.3)	1 (2.8)
p – Value ^‡^	0.500 ^‡‡^		0.67 ^‡‡^		0.433 ^‡‡^		0.545 ^‡‡^		0.478 ^‡‡^		0.455 ^‡‡^	
**Total SDQ** ^§§^												
Normal	25 (73.5)	31 (91.2)	30 (75.0)	26 (92.9)	27 (73.0)	29 (93.5)	25 (69.4)	31 (96.9)	21 (63.6)	35 (100.0)	20 (62.5)	36 (100.0)
Altered ^||^	9 (26.5)	3 (8.8)	10 (25.0)	2 (7.1)	10 (27.0)	2 (6.5)	11 (30.6)	1 (3.1)	12 (36.4)	0 (0.0)	12 (37.5)	0 (0.0)
p – Value ^‡^	**0.056** ^§^		**0.054** ^‡‡^		**0.027** ^§^		**0.003** ^§^		**<0.001** ^§^		**<0.001** ^§^	

^*^ PedsQL = Pediatric Quality of Life Inventory;

^†^ N(%) = Number of participants and percentage of participants;

^¶^ CCEB = *Critério de Classificação Econômica Brasil* ;

^**^ A1 = Class A1;

^††^ B1/B2 = Class B1/B2;

^§§^ SDQ = The Strengths and Difficulties Questionnaire

^||^ Linear-by-linear association;

^‡^ p – Value;

^§^ Pearson’s Chi-square;

^‡‡^ Fisher’s Exact;

**Table 2:** - Bivariate analysis of the association between PedsQL ^*^ variables (parents and adolescents) and selected variables (n = 68). Belo Horizonte, MG, Brazil, 2021

**Characteristics**	**Parents** **Dimensions**						**Adolescents** **Dimensions**					
	**Physical**		**Psychosocial**		**General quality of life**		**Physical**		**Psychosocial**		**General quality of life**	
	**high**	**low**	**high**	**low**	**high**	**low**	**high**	**low**	**high**	**low**	**high**	**low**
	**n(%) ^†^**	**n(%) ^†^**	**n(%) ^†^**	**n(%) ^†^**	**n(%) ^†^**	**n(%) ^†^**	**n(%) ^†^**	**n(%) ^†^**	**n(%) ^†^**	**n(%) ^†^**	**n(%) ^†^**	**n(%) ^†^**
**Gender**												
Female	30 (65.2)	9 (40.9)	18 (62.1)	21 (53.8)	19 (63.3)	20 (52.6)	12 (75.0)	27 (51.9)	28 (66.7)	11 (42.3)	23 (69.7)	16 (47.1)
Male	16 (34.8)	13 (59.1)	11 (37.9)	18 (46.2)	11 (36.7)	18 (47.4)	4 (25.0)	25 (48.1)	14 (33.3)	15 (57.7)	10 (30.3)	18 (52.9)
p – Value ^‡^	0.058 ^§^		0.498 ^§^		0.376 ^§^		0.103 ^§^		0.048 ^§^		0.060§	
**Age (years old)**												
11	14 (30.4)	6 (27.3)	9 (31.0)	11 (28.2)	9 (30.0)	11 (28.9)	5 (31.3)	15 (28.8)	13 (31.0)	7 (26.9)	12 (36.4)	8 (23.5)
12	9 (19.6)	7 (31.8)	5 (17.2)	11 (28.2)	6 (20.0)	10 (26.3)	2 (12.5)	14 (26.9)	9 (21.4)	7 (26.9)	5 (15.2)	10 (29.5)
13	13 (28.3)	4 (18.2)	6 (20.7)	11 (28.2)	7 (23.3)	10 (26.3)	5 (31.3)	12 (23.1)	11 (26.2)	6 (23.1)	9 (27.3)	8 (23.5)
14	10 (21.7)	5 (22.7)	9 (31.0)	6 (15.4)	8 (26.7)	7 (18.5)	4 (25.0)	11 (21.2)	9 (21.4)	6 (23.1)	7 (21.2)	8 (23.5)
p – Value ^‡^	0.867 ^||^		0.451 ^||^		0.653 ^||^		0.678 ^||^		0.882 ^||^		0.623||	
**School grade**												
8 ^th^	1 (2.2)	0 (0.0)	0 (0.0)	1 (2.6)	1 (3.3)	10 (26.3)	1 (6.3)	0 (0.0)	1 (2.4)	0 (0.0)	1 (3.0)	0 (0.0)
9 ^th^	13 (28.3)	8 (36.4)	9 (31.0)	12 (30.8)	9 (30.0)	12 (31.6)	5 (31.3)	16 (30.8)	12 (28.6)	9 (34.6)	11 (33.3)	10 (29.4)
1 ^st^ grade HS ^¶^	11 (23.9)	5 (22.7)	6 (20.7)	10 (25.6)	6 (20.0)	10 (26.3)	1 (6.3)	15 (28.8)	10 (23.8)	6 (23.1)	6 (18.2)	9 (26.5)
2 ^nd^ grade HS ^¶^	10 (21.7)	4 (18.2)	5 (17.3)	9 (23.1)	5 (16.7)	9 (23.9)	3 (18.8)	11 (21.2)	8 (19.0)	6 (23.1)	6 (18.2)	8 (23.5)
3 ^rd^ grade HS ^¶^	11 (23.9)	5 (22.7)	9 (31.0)	7 (17.9)	9 (30.0)	7 (18.4)	6 (37.5)	10 (19.2)	11 (26.2)	5 (19.2)	9 (27.3)	7 (20.6)
p – Value ^‡^	0.754||		0.389 ^||^		0.704 ^||^		−		0.707 ^||^		0.947||	
**CCEB** ^**^												
A1 ^††^	28 (60.9)	12 (54.5)	17 (58.6)	23 (59.0)	19 (63.3)	21 (55.3)	12 (75.0)	28 (53.8)	27 (64.3)	13 (50.0)	22 (66.7)	17 (50.0)
B1/B2 ^‡‡^	18 (39.1)	10 (45.5)	12 (41.4)	16 (41.0)	11 (36.7)	17 (44.7)	4 (25.0)	24 (46.2)	15 (35.7)	13 (50.0)	11 (33.3)	17 (50.0)
p – Value ^‡^	0.620 ^§^		0.977 ^§^		0.502 ^§^		0.133		0.245 ^§^		0.167§	
**Self-perceived health**												
Poor/Average	12 (26.1)	1 (4.5)	7 (24.1)	6 (15.4)	9 (30.0)	4 (10.5)	8 (50.0)	5 (9.6)	11 (26.2)	2 (7.7)	10 (30.3)	3 (8.8)
Good/Excellent	34 (73.9)	21 (95.5)	22 (75.9)	33 (84.6)	21 (70.0)	34 (89.5)	8 (50.0)	47 (90.4)	31 (73.8)	24 (92.3)	23 (69.7)	31 (91.2)
p – Value ^‡^	0.031 ^§§^		0.364 ^§§^		0.043 ^§§^		0.001 ^§§^		0.054 ^§§^		0.026§	
**Learning Goal**												
< 31	30 (65.2)	13 (59.1)	20 (69.0)	14 (50.0)	20 (66.7)	23 (60.50)	10 (62.5)	33 (63.5)	28 (66.7)	15 (57.7)	22 (66.7)	20 (58.8)
≥ 31	16 (34.8)	9 (40.9)	9 (31.0)	14 (50.0)	10 (33.3)	15 (39.5)	6 (37.5)	19 (36.5)	14 (33.3)	11 (42.3)	11 (33.3)	14 (41.2)
p – Value ^‡^	0.624 ^§^		0.398 ^§^		0.602 ^§^		0.944 ^§^		0.456 ^§^		0.507§	
**Performance-approach Goal**												
< 15	21 (45.7)	18 (81.8)	17 (58.6)	22 (56.4)	17 (56.7)	22 (57.9)	10 (62.5)	29 (55.8)	23 (54.8)	16 (61.5)	19 (57.6)	20 (58.8)
≥ 15	25 (54.3)	4 (18.2)	12 (41.4)	17 (43.6)	13 (43.3)	16 (42.1)	6 (37.5)	23 (44.2)	19 (45.2)	10 (38.5)	14 (42.4)	14 (47.2)
p – Value ^‡^	0.005 ^§^		0.855 ^§^		0.919 ^§^		0.634 ^§^		0.583 ^§^		0.918§	
**Performance-avoidance Goal**												
≤ 8	22 (47.8)	10 (45.5)	14 (48.3)	18 (46.2)	14 (46.7)	18 (47.4)	4 (25.0)	28 (53.8)	16 (38.1)	16 (61.5)	9 (27.3)	22 (64.7)
> 8	24 (52.2)	12 (54.5)	15 (51.7)	21 (53.8)	16 (53.3)	20 (52.6)	12 (75.0)	24 (46.2)	26 (61.9)	10 (38.5)	24 (72.7)	12 (35.3)
p – Value ^‡^	0.855 ^§^		0.862 ^§^		0.954 ^§^		0.043 ^§^		0.060 ^§^		0.002§	
**Prosocial Behavior SDQ** ^||||^												
Normal	46 (100.0)	21 (95.5)	29 (100.0)	38 (97.4)	30 (100.0)	37 (97.4)	16 (100.0)	51 (98.1)	41 (97.6)	26 (100.0)	32 (97.0)	34 (100.0)
Altered ^||^	0 (0.0)	1 (4.5)	0 (0.0)	1 (2.6)	0 (0.0)	1 (2.6)	0 (0.0)	1 (1.9)	1 (2.4)	0 (0.0)	1 (3.0)	0 (0.0)
p – Value ^‡^	0.324 ^§§^		0.574 ^§^		0.559 ^§§^		0.765 ^§§^		0.618 ^§§^		0.493§§	
**Total SDQ** ^||||^												
Normal	40 (87.0)	19 (86.4)	23 (79.3)	36 (92.3)	23 (76.7)	36 (94.7)	12 (75.0)	47 (90.4)	33 (78.6)	26 (100.0)	25 (75.8)	33 (97.1)
Altere ^||^	6 (13.0)	3 (13.6)	6 (20.7)	3 (7.7)	7 (23.3)	2 (5.3)	4 (25.0)	5 (9.6)	9 (21.4)	0 (0.0)	8 (24.2)	1 (0.0)
p – Value ^‡^	0.610 ^§§^		0.115 ^§§^		0.034 ^§§^		0.124 ^§§^		0.009 ^§§^		0,012§§	

^*^ PedsQL = Pediatric Quality of Life Inventory;

^†^ N(%) = Number of participants and percentage of participants;

^¶^ HS = High School;

^**^ CCEB = *Critério de Classificação Econômica Brasil* ;

^††^ A1 = Class A1;

^‡‡^ B1/B2 = Class B1/B2;

^§^ Pearson’s Chi-square;

^||||^ SDQ = The Strengths and Difficulties Questionnaire

^||^ Linear-by-linear association;

^‡^ p – Value;

^§§^ Fisher’s Exact;

**Table 3:** - Results of multivariate logistic regression analyzes between quality of life (PedsQL ^*^ ) according to parents and adolescents (n = 68). Belo Horizonte, MG, Brazil, 2018 and 2021

**Characteristics**		**Model 1** ^†^ **- General Quality of Life Parents 2018**				**Model 2** ^‡^ **- General Quality of Life Adolescents 2018**			
		Initial model		Final model		Initial model		Final model	
		OR (95%CI) ^§^	p-Value ^||^	OR (95%CI) ^§^	p-Value ^||^	OR (95%CI) ^§^	p-Value ^¶^	OR (95%CI) ^§^	p-Value ^¶^
School Grade	6 ^†^	1.92 (0.47-7.90)	0.368	−	−	4.31 (0.99-18.78)	0.052	**4.50 (1.06-19.04)**	**0.041**
	7 ^†^	0.85(0.20-3.58)	0.826	−	−	11.06 (2.15-56.72)	0.004	**9.75 (1.98-47.94)**	**0.005**
	8 ^†^	0.41 (0.08-1.97)	0.264	−	−	2.28 (0.48-10.76)	0.299	2.65(0.57-11.99)	0.213
Self-perceived health		−	−	−	−	−	−	−	−
Learning Goal		−	−	−	−	2.07 (0.66-6.53)	0.212	−	−
Performance-avoidance Goal		−	−	−	−	1.61 (0.54-4.82)	0.392		
Prosocial Behavior SDQ ^**^		−	−	−	−	−	−	−	−
SDQ ^**^ (total)		6.21 (1.16-33.16)	0.033	**5.35 (1.06-26.9)**	**0.042**	−	−	−	−
**Characteristics**		**Model 3** ^††^ **- General Quality of Life Parents 2021**				**Model 4** ^‡‡^ **- General Quality of Life Adolescents 2021**			
		Initial model		Final model		Initial model		Final model	
		OR (95%CI) ^§^	p-Value ^§§^	OR (95%CI) ^§^	p-Value ^§§^	OR (95%CI) ^§^	p-Value ^||||^	OR (95%CI) ^§^	p-Value ^||||^
Gender		−	−	−	−	1.78 (0.56-5.62)	0.325		
CCEB ^¶¶^						2.26 (0.71-7.19)	0.167	−	−
Self-perceived health		2.93 (0.76-11.29)	0.118	−	−	2.52 (0.51-12.38)	0.256	−	−
Performance-avoidance Goal		−	−	−	−	4.44 (1.39-14.11)	0.011	**5.02 (1.67-15.12)**	**0.004**
Prosocial Behavior SDQ ^**^		−	−	−	−	−	−	−	−
SDQ ^**^ (total)		4.36(0.79-23.96)	0.090	**5.51 (1.05-28.98)**	**0.044**	7.83 (0.78-78.58)	0.080	**11.05 (1.18-103.77)**	**0.035**

^*^ PedsQL = Pediatric Quality of Life Inventory;

^†^ Model 1 = 9 ^th^ grade, Total SDQ: Altered, Poor/average self-perceived health was not included in the model as it did not converge due to the category without observations, Age-adjusted Final Model;

^‡^ Model 2 = 9 ^th^ grade, Poor/average self-perceived health, Learning Goal and Performance-avoidance Goal: highest score, SDQ was not included in the model as it did not converge due to the category without observations;

^||^ Wald test, Adjustment of initial/final models (Hosmer and Lemeshow): p=0.638/p=0.576;

^¶^ Wald test, Adjustment of initial/final models (Hosmer and Lemeshow): p=0.639/p=1.000;

^††^ Model 3 = Poor/average self-perceived health, Total SDQ: Altered, Age-adjusted Final Model;

^‡‡^ Model 4 = Male, Poor/average self-perceived health, Performance-avoidance Goal: highest score, Total SDQ: Altered;

^§^ OR(95%CI) = Odds Ratio and 95% confidence interval;

^§§^ Wald test, Adjustment of initial/final models (Hosmer and Lemeshow): p=0.713/p=0.834;

^||||^ Wald test, Adjustment of initial/final models (Hosmer and Lemeshow): p=0.700/p=0.289;

^¶¶^ CCEB = *Critério de Classificação Econômica Brasil*

^**^ SDQ = The Strengths and Difficulties Questionnaire;

In summary, considering the logistic regression analyzes in both periods, the variable Strengths and Difficulties, measured by the total SDQ score, tested in models 1 and 3, was associated with quality of life in the parents’ assessment in 2018 and 2021. According to the adolescents’ perception, quality of life was associated, in 2018, with school grade, and in 2021 with Performance-avoidance Goal and total SDQ.

## Discussion

 Health and the social determinants of health find, in the school environment, a field of study and reflection, as school age is a crucial period of physical and cognitive growth ^(^ ^27^ ^)^ . As the research context was a privately financed institution, the participants belonged to higher strata in terms of economic class. In the comparison between the periods 2018 and 2021, there was a worsening in the assessment of self-perceived health, as 8.8% and 19.2%, respectively, considered their health to be regular, poor or very poor, considering the emergency situation in public health. The suspension of face-to-face classes and the adaptation of routine and school cycles to the remote context caused insecurity and uncertainty, and social isolation, with reduced contact with peers, affected the well-being of adolescents ^(^ ^17^ ^-^ ^18^ ^)^ . The school is a collective space favorable to health promotion, which offers children and adolescents a field to raise awareness and practice healthy lifestyle habits, self-care and risk prevention ^(4,15).^

 Since adolescence is a critical and sensitive phase in the lives of individuals and their families, when profound physical, emotional ^(^ ^28^ ^)^ , social and cognitive transformations are experienced, it is important to monitor health and well-being from different aspects, with quality of life a very appropriate indicator ^(^ ^29^ ^)^ . Regarding the assessment of quality of life in the perception of parents, the increase in the psychosocial dimension score (emotional, social and school) evidenced by the study can be explained by the use of technological resources accessible to the social stratum studied, and the decrease in the physical dimension score reflects the health and epidemiological crisis that occurred as a result of the spread of SARS-CoV-2. In addition to the damage in the educational sphere, the closure of schools generated a significant change in the dynamics of families, who needed to reconcile the professional commitments of parents with the student demands of adolescents in larger spaces of shared time ^(^ ^30^ ^)^ , which involved greater interaction between parents in the school life of adolescents ^(^ ^14^ ^)^ . When observing the quality of life based on the adolescents’ self-report, the decrease in scores in the psychosocial dimension can be justified by the high demand for interaction with peers inherent to the adolescence phase, which was hampered by the necessary social distancing measures. Interaction and communication are known to be natural needs of human beings ^(^ ^10^ ^)^ and the school environment is a place for growth, development of cognitive and behavioral skills and socialization with repercussions on the quality of life of children and adolescents ^(^ ^8^ ^,^ ^31^ ^)^ . 

 In addition, associated to quality of life, aspects such as gender, father’s and mother’s education and family income were investigated in a study carried out with Iranian adolescents aged between 15 and 18, which showed significant effects of socioeconomic inequality on quality of life related to the health of Iranian adolescents, indicating the need for public policies in favor of a healthy society for a better quality of life ^(^ ^29^ ^)^ . 

 In the analysis of motivation to learn, in the comparison between the periods studied, a drop in the Learning Goal and an increase in the Performance-avoidance Goal was evidenced, conditions determined, possibly, by pedagogical reasons, with the sudden implementation of a remote teaching model. Adapting the educational system, involving teachers and students around content, teaching, learning and assessment methods, is one of the challenges of this historical moment, as the motivation of students to learn also involves teaching, structural and social issues ^(^ ^32^ ^)^ . With regard to the insertion of information and communication technologies, a survey of Brazilian High School students revealed that 20% of participants denied the sufficiency of remote classes. The main reason was the difficulty in understanding the content transmitted, a result that makes us think about remote interaction between teacher and student and how motivating and effective it can be for basic education ^(^ ^33^ ^)^ . 

 The interruption of the face-to-face school routine also had an impact on the interest of adolescents at a public institution in the southeast region of Brazil - a region that does not have the highest percentages of school exclusion in the country -, where almost 40% of students indicated a lack of interest in distance school activities and others 49% expressed a tendency to school evasion ^(^ ^2^ ^)^ . Concerning the segment of the final grades of Elementary Education, the results of another research carried out during the pandemic period showed that students who were usually intrinsically motivated to learn, were extrinsically motivated or unmotivated with regard to remote teaching, suggesting direct implications between the quality of engagement between teacher and student and motivation to learn in this teaching modality ^(^ ^34^ ^)^ . For university students, resuming face-to-face activities represented an improvement in motivational levels for studying, permeated by social contact ^(^ ^35^ ^)^ . 

 In this sense, the odds ratio values found in the multivariate logistic regression analyzes carried out with the sample of the present study showed interesting results. The greater chances of having a better self-reported quality of life found among adolescents in the initial grades of Elementary Education II corroborate pre-pandemic studies ^(^ ^36^ ^-^ ^37^ ^)^ , which showed that younger students have a better general quality of life when compared to students in the final grades in different educational strata. A longitudinal investigation carried out in 2018 with Chinese adolescent students showed a drop in life satisfaction and a gradual increase in hopelessness in the academic career, associated with individual and contextual aspects, such as resilience, sociocultural factors and family environment ^(^ ^38^ ^)^ . 

 In both periods investigated, the adolescent’s typical behavior favored them to have a good or excellent quality of life in the perception of their parents and in their own assessment, in which having a normal result in the analysis of strengths and difficulties, related to the mental health of the adolescent student, increased the chances of having a better quality of life by more than five times. This data suggests a rapprochement between the constructs of mental health and quality of life. The joint action of health and education professionals at school contributes to the implementation and development of actions for adolescents in search of healthy behavior towards themselves, their activities and the group they are part of ^(^ ^3^ ^,^ ^39^ ^-^ ^40^ ^)^ . 

 Thus, it is also important to highlight the link between physical and psychosocial health and the motivational behavior adopted by adolescents towards learning, as students who presented adequate results in the analysis of strengths and difficulties and a better quality of motivation to learn, indicated by low learning avoidance, were between five and eleven times more likely to have a better general quality of life in the analysis of the pandemic period. Health, in its multiple dimensions, dialogues with appropriate conduct in the school environment, including the fulfillment of academic tasks. The presence of risk factors for mental health problems, for example, affected the health-related quality of life of German adolescents, as shown in a longitudinal study in 2017 ^(^ ^41^ ^)^ , in line with the negative impact on quality of life observed in young Brazilian university students with symptoms of depression, anxiety and stress ^(^ ^42^ ^)^ . 

 Psychological conditions and emotional disorders are not rare among adolescents ^(^ ^43^ ^)^ , and the pandemic caused by SARS-CoV-2 contributed to the epidemiology of these disorders. According to Chinese studies carried out in 2020 with adolescents aged 12 to 18 and young people, the prevalence of depressive symptoms, anxiety, post-traumatic stress and other psychological problems is high. Access to information about the disease and prevention measures was considered a protective factor against the development of emotional symptoms ^(^ ^19^ ^-^ ^20^ ^)^ . Likewise, research conducted with children and adolescents in Germany in 2020 ^(^ ^21^ ^)^ and in Brazil in 2022 ^(^ ^18^ ^)^ indicated that two thirds of participants reported being highly overwhelmed, and that adolescents expressed increased feelings of loneliness, anxiety and sadness due to the pandemic. Self-reported health-related quality of life was significantly lower when compared to the period before the health crisis, in addition to reporting more mental health problems and higher levels of anxiety. Furthermore, children and adolescents with lower socioeconomic status were more impacted, reinforcing the need to implement health promotion and disease prevention strategies to maintain mental health and improve the quality of life of children and adolescents, especially those who are in a situation of greater vulnerability ^(^ ^44^ ^)^ . 

The present study highlighted pedagogical, education, physical and socio-emotional health repercussions for adolescents who were in their final grades of Elementary Education during the period of the pandemic caused by SARS-CoV-2. The loss of follow-up of part of the group researched in 2018 constituted a limitation of the research. As this is a sample from a single social stratum, students from a privately financed institution, the results do not represent the massive Brazilian reality, and further research is desirable to expand the conclusions.

The contributions of this work to the advancement of scientific knowledge in the health area encompass a range of factors, intrinsic and extrinsic to the individual, inherent to the development of childhood and adolescence, in which intersectoral action between health services and educational institutions can favor the early detection of diseases, promoting health in its broadest sense and constituting a significant field of activity for Nursing professionals.

## Conclusion

The majority of participants were female and belonged to class A. In the comparison between 2018 and 2021, there was a worsening in the assessment of self-perceived health. The average responses regarding the value attributed to health remained stable in both periods. In the assessment of QoL by parents, there was a statistically significant difference between the scores of the social and psychosocial dimensions, both with higher scores in 2021. Among adolescents, there were differences between QoL scores in the physical, social, school, emotional and psychosocial dimensions. For strengths and difficulties, there was no significant difference in the percentage of responses between the categories in the two steps of the study. Adolescents with a normal score in the mental health assessment had a greater chance of having a better quality of life during the periods investigated (OR=5.35 and OR=5,51). Students in the initial grades of Elementary Education II showed more motivation to learn, increasing the chance of improving self-reported quality of life in the two periods, respectively, by up to 9.7 and 5.02 times.

In summary, the association between health, quality of life, behavior and motivation to learn among adolescent students raises important reflections on approaches to adolescent health in the school environment. The urgency of a broader look at this space stands out, which must fulfill important functions beyond curricular educational ones, attributing meanings and building an increasingly effective and comprehensive education.
