# Supplementary material for: Quality of life, health perception, learning motivation and behavior of adolescents in an educational institution
Source: Rev Lat Am Enfermagem. 2024 Oct 25;32:e4338. doi: 10.1590/1518-8345.6919.4338 (PMC11526294; doi:10.1590/1518-8345.6919.4338)
Supplement: Translation

# Translation: Calidad de vida, percepción de la salud, motivación para el aprendizaje y comportamiento de adolescentes en una institución educativa ^*^

**DOI:** 10.1590/1518-8345.6919.4337

**Authors:** Graziela Nunes Alfenas Fernandes, Stela Maris Aguiar Lemos

**Affiliations:** ^1^ Universidade Federal de Minas Gerais, Faculdade de Medicina, Belo Horizonte, MG, Brasil.; ^2^ Becaria de la Coordenação de Aperfeiçoamento de Pessoal de Nível Superior (CAPES), Brasil.

**Keywords:** Adolescente, Salud, Calidad de Vida, Comportamiento, Aprendizaje a Distancia, Percepción

## Abstract

**Objetivo:**

analizar variables sociodemográficas, calidad de vida, autopercepción de la salud, motivación para el aprendizaje y comportamiento de adolescentes en 2018 y 2021.

**Método:**

estudio transversal observacional con 124 adolescentes en 2018 y 68 en 2021. Para la recolección de datos se utilizó un formulario para las variables sociodemográficas, Cuestionario de Calidad de Vida Pediátrica, instrumento de Autopercepción de la Salud, Escala de Motivación para el Aprendizaje y Cuestionario de Capacidades y Dificultades. La recolección de datos se realizó mediante formularios en *Google Forms* . Para el análisis se utilizó estadística descriptiva y regresión logística.

**Resultados:**

la mayoría de los participantes pertenecían a la clase A. En la comparación entre 2018 y 2021, hubo un empeoramiento en la evaluación de la autopercepción de la salud. En la evaluación de la CV por parte de los padres, hubo diferencia estadísticamente significativa entre las puntuaciones de las dimensiones social y psicosocial. Entre los adolescentes, hubo diferencias entre las puntuaciones de la CV en las dimensiones física y psicosocial.

**Conclusión:**

el adolescente con mejor evaluación de la salud mental tuvo mayores posibilidades de tener una mejor calidad de vida en los períodos investigados (OR=5,35 y OR=5,51). Los estudiantes más jóvenes mostraron una mayor motivación para aprender, aumentando la posibilidad de mejorar su calidad de vida hasta 9,75 y 5,02 veces en los dos periodos, respectivamente.

## Destacados

**(1)** El ambiente escolar es un espacio propicio para la promoción de la salud física, mental y social.

**(2)** Durante la adolescencia se debe concienciar sobre hábitos de vida saludables.

**(3)** Los aspectos relacionados con la salud pueden influir en el comportamiento y el aprendizaje.

**(4)** La calidad de vida (CV) y sus dimensiones están relacionadas con la motivación para aprender.

**(5)** La salud mental del adolescente está relacionada con la autopercepción de la CV.

## Introducción

 Una importante crisis de Salud Pública trajo cambios globales significativos entre 2019 y 2021 en términos de la forma en que las personas viven y coexisten, creando la necesidad inmediata de adaptaciones de todo tipo. La pandemia causada por el Síndrome Respiratorio Agudo Severo coronavirus 2 (SARS-CoV-2) cambió la forma de pensar y practicar la educación en Brasil y en el mundo, afectando a más del 90% de los estudiantes en todo el planeta y a los procesos educativos en más de 180 países. En Brasil, las medidas de distanciamiento social para mitigar el virus resultaron en la suspensión de actividades escolares presenciales y decretos gubernamentales autorizaron la Enseñanza Remota de Emergencia (ERE), implementando repentinamente actividades escolares por medio de tecnologías de la información y la comunicación ^(^ ^1^ ^-^ ^3^ ^)^ . 

 El individuo, durante la niñez y la adolescencia, encuentra en la escuela una dimensión importante de su calidad de vida, pues en este espacio, además de adquirir conocimientos curriculares, desarrolla valores fundamentales para su formación integral como ser humano y ciudadano. En este sentido, el ambiente escolar es propicio para la realización de programas efectivos e integrales de promoción y educación en salud ^(^ ^4^ ^)^ , ya que, por medio de la intersectorialidad ^(^ ^5^ ^-^ ^6^ ^)^ , coopera para construir conocimientos sobre la naturaleza humana y su desarrollo, mejorando habilidades y creando aptitudes para el autocuidado, el cuidado del medio ambiente y el bienestar general ^(^ ^7^ ^-^ ^9^ ^)^ , y reúne demandas cognitivas, físicas, emocionales, familiares y socioeconómicas de los estudiantes hacia la salud integral. 

 Así, destacando la escuela como lugar de cuidado, estimulación y crecimiento en los diferentes ámbitos de la vida, el nuevo formato de construcción del conocimiento trajo reflexiones sobre características fundamentales de este proceso didáctico-pedagógico, involucrando equipos técnicos, docentes y estudiantiles en torno a la enseñanza y al aprender ^(^ ^10^ ^)^ . Se sabe que la motivación humana para realizar tareas sigue estímulos internos y aspectos contextuales. Durante la adolescencia, un período caracterizado por transiciones, la motivación es esencial para que los jóvenes puedan alcanzar sus objetivos y experimentar un desarrollo saludable, incluidas las necesidades psicológicas, la autodisciplina y el estímulo académico ^(^ ^11^ ^)^ . En el ámbito del aprendizaje, la motivación puede estar enfocada fundamentalmente a la mejora individual o dirigida principalmente al desempeño en la realización de actividades académicas ^(^ ^12^ ^)^ . Combinados con la motivación para aprender, factores relacionados con la salud mental ^(^ ^13^ ^)^ , comportamiento, aspectos familiares ^(^ ^14^ ^)^ y las cuestiones socioeconómicas y demográficas ^(^ ^15^ ^)^ pueden influir en el proceso educativo de los adolescentes ^(^ ^2^ ^)^ . 

 Estudios realizados en Brasil ^(^ ^16^ ^-^ ^18^ ^)^ y en países como China y Alemania ^(^ ^19^ ^-^ ^21^ ^)^ mostraron un empeoramiento de la salud mental, la autoestima y el bienestar psicosocial de los adolescentes durante la pandemia. Los efectos negativos impactaron el desarrollo y el desempeño en diferentes áreas de la vida. También vale la pena destacar las consecuencias de estos factores para la calidad de vida general y sus dimensiones física, emocional, escolar y social en esta población, resaltadas por la implementación de nuevas estrategias pedagógicas y didácticas inherentes al modelo educativo de emergencia. 

Así, pensando en la educación básica y rescatando la enseñanza y el aprendizaje como procesos de interacción que permean el vínculo, el desarrollo biopsicosocial y la escuela, el objetivo principal de este estudio fue analizar variables sociodemográficas, calidad de vida, autopercepción de la salud, motivación para el aprendizaje y comportamiento de adolescentes de una institución privada antes de la pandemia de COVID-19, en 2018, y durante, en 2021.

## Método

### Diseño del estudio y definición de la muestra

Se trata de un estudio observacional cuantitativo de corte transversal con una muestra estratificada por género, edad y año escolar. La muestra estuvo compuesta por 124 adolescentes matriculados en la Educación Primaria II de una escuela brasileña con financiación privada antes de la pandemia de COVID-19 en 2018, y 68 estudiantes durante la pandemia de COVID-19 en 2021, donde estaban matriculados aproximadamente 244 estudiantes en el segmento educativo investigado.

 El cálculo de la muestra consideró un error muestral del 5%, un intervalo de confianza del 95% y una prevalencia del 15% considerando la calidad de vida como resultado de interés, estimándose una muestra de 114 adolescentes seleccionados mediante muestreo aleatorio estratificado por género y año escolar. Se utilizó una prueba para estimar una proporción en el *software* *Minitab 14 Release* . 

 Para la motivación para el aprendizaje, un tamaño de muestra de 124 individuos obtuvo un poder estadístico del 80% en la estimación de la baja motivación para aprender, considerando como parámetro el 22,5% en la población. La precisión obtenida de este tamaño de muestra y poder estadístico es del 10% y el nivel de significancia fue del 5%. Se utilizó una prueba para estimar una proporción en el *software* *Minitab 17* . 

### Criterios de selección

 Como criterios de inclusión, en el año 2018 se consideraron adolescentes matriculados en el segmento de Educación Primaria II de la institución investigada, con edades comprendidas entre 11 y 14 años y estudiantes del 6º al 9º año escolar, siendo incluidos 124 adolescentes. Se utilizó como criterio de exclusión la no cumplimentación de los instrumentos de investigación. En 2021, los 124 participantes incluidos en 2018 fueron invitados a participar en la segunda etapa del estudio, 56 se negaron por motivos como cambio de escuela, falta de tiempo para completar los instrumentos de investigación y cambio de contacto telefónico, y 68 estuvieron de acuerdo con la nueva recolección de datos ( Figura 1 ). 

**Figura 1:** - Flujograma de los participantes de la investigación 2018-2021

^*^ EPII = Educación Primaria II; ^†^ ES = Educación Secundaria

### Aspectos éticos

 Esta investigación fue aprobada por el Comité de Ética en Investigación de la *Universidade Federal de Minas Gerais* (UFMG) bajo Certificado de Presentación de Apreciación Ética (CAAE, por sus siglas en portugués) número 80162417.1.0000.5149. El proyecto fue aprobado bajo dictamen número 2.422.795 en diciembre de 2017 y la modificación del proyecto fue aprobada bajo dictamen número 4.446.496 en diciembre de 2020. En 2018, los padres o tutores y los adolescentes participantes firmaron el Formulario de Consentimiento Libre e Informado (TCLE, por sus siglas en portugués) y el Formulario de Asentimiento Libre e Informado (TALE, por sus siglas en portugués), respectivamente. En 2021, debido a la enseñanza remota de emergencia, los participantes aceptaron el TCLE y el TALE por medio de formularios electrónicos. 

### Variables del estudio

 Para este estudio se consideraron informaciones de caracterización de los participantes - género, edad, año escolar y clasificación económica (CCEB) -, autopercepción de la salud y capacidades y dificultades (SDQ – Por) como variables explicativas, y calidad de vida, autorreporte y reporte de los padres en las dimensiones física, psicosocial y calidad de vida general (PedsQL ^TM^ ), y motivación para aprender, considerados sus tres dominios (EMAPRE), como variables respuesta. 

### Instrumentos utilizados para recolectar la información

 En este estudio se utilizaron cinco instrumentos: cuestionario que contiene las variables sociodemográficas de los participantes, el Cuestionario de Calidad de Vida Pediátrica – PedsQL™ 4.0, el cuestionario sobre la Autopercepción de la Salud, la Escala de Motivación para el Aprendizaje – EMAPRE y el *The Strengths and Difficulties Questionnaire* – SDQ. 

 Formulario para obtener variables sociodemográficas (género, edad y año escolar y clasificación económica). Para clasificar la clase económica, se adoptó el *Critério de Classificação Econômica Brasil* - CCEB ^(^ ^22^ ^)^ . Para ello, se solicitó a los padres y/o tutores de los adolescentes llenar los ítems referentes al poder adquisitivo y nivel de educación del jefe de familia, solamente en el año 2018, para luego agrupar a los participantes en las clases de A a E. 

 Cuestionario de Calidad de Vida Pediátrica - PedsQL™ 4.0. Este instrumento fue estandarizado y validado para el portugués brasileño ^(^ ^23^ ^-^ ^24^ ^)^ , y permite evaluar la calidad de vida (CV) en cuatro dominios: físico (dimensión física), emocional, social y escolar (dimensión psicosocial). Para su uso se solicitó permiso a los autores. Además de la versión destinada a los padres para que la completen, este cuestionario también tiene una versión destinada a los adolescentes, para el autorreporte de la calidad de vida, y ambas fueron utilizadas en la investigación. Para obtener la puntuación se invierten los ítems y se transforman linealmente en una escala de 0 a 100: 0 - 100; 1 - 75; 2 - 50; 3 - 25; 4 - 0. Luego, las puntuaciones de cada ítem se suman y se dividen por el número de puntuaciones respondidas, presentando el promedio de cada ítem del dominio. Para obtener datos de la Dimensión Psicosocial se suman las puntuaciones de las escalas Emocional, Social y Escolar. Para la Dimensión Física se utilizan las respuestas dadas en la escala física. Para la puntuación global, el cálculo se realiza dividiendo la suma de todos los ítems por el número de ítems respondidos en todas las escalas. 

Cuestionario sobre la Autopercepción de la Salud. Este cuestionario fue preparado por las investigadoras con las siguientes preguntas: “¿Cómo evalúa/considera actualmente su salud?” y “¿Cómo calificaría su salud?”. Las respuestas a la primera pregunta se evalúan mediante una escala Likert con las opciones: muy mala, mala, regular, buena y excelente. Para la segunda pregunta se utilizó una escala numérica de 0 a 10 para asignar la respuesta, considerándose cero muy mala y 10 excelente.

 Escala de Motivación para el Aprendizaje – EMAPRE ^(^ ^12^ ^)^ . Este instrumento permite a los estudiantes evaluar su motivación para estudiar y realizar actividades académicas. La escala consta de 28 preguntas distribuidas en tres dominios: Meta Aprender, Meta Desempeño-aproximación y Meta Desempeño-evitación. La Meta Aprender es aquella en la que el estudiante busca desafíos y los utiliza como recurso para su propio aprendizaje y desarrollo intelectual. La Meta Desempeño-aproximación resalta la preocupación del estudiante principalmente por superar a los demás demostrando su propia capacidad. Y la Meta Desempeño-evitación se refiere a la condición en la que el estudiante evita situaciones en las que pueda ocurrir fracaso en la realización de las tareas escolares propuestas. Así, todas las preguntas se relacionan con la motivación, la actitud y los objetivos respecto al aprendizaje. EMAPRE es una escala tipo Likert, con opciones de respuesta “de acuerdo”, “no sabe” y “en desacuerdo”. Este instrumento fue desarrollado con estudiantes de Educación Secundaria y fue sometido a análisis factorial en el que los tres factores explicaron el 40,56% de la varianza total en el análisis de los componentes principales, y los tres dominios presentaron Alfa de Cronbach α=0,795, α=0,798 y α=0,801, respectivamente, en el análisis de consistencia interna, destacando la idoneidad del instrumento para la muestra de adolescentes propuesta en el estudio ^(^ ^25^ ^)^ . 

 Cuestionario de Capacidades y Dificultades (SDQ) ^(^ ^26^ ^)^ . Este instrumento fue construido en 1997 por Goodman y validado en Brasil por Fleitlich, Cortázar y Goodman en 2000. El SDQ es un cuestionario que permite realizar un seguimiento de los problemas de salud mental infantojuvenil. El cuestionario consta de 25 ítems, distribuidos en cinco escalas, a saber: síntomas emocionales (cinco ítems); problemas de comportamiento (cinco ítems); hiperactividad/falta de atención (cinco ítems); problemas de relación (cinco ítems) y comportamiento prosocial (cinco ítems). De estos 25 ítems, 10 son sobre capacidades, 14 sobre dificultades y uno de ellos se considera neutral. Cada uno de los ítems se puede responder como “falso”, “más o menos verdadero” o “verdadero”. La puntuación de cada una de las escalas se obtiene sumando las puntuaciones de los 5 ítems, generando una puntuación que varía de 0 a 10. Las puntuaciones de las escalas de hiperactividad, síntomas emocionales, problemas de conducta y problemas de relación con pares se suman para generar una puntuación total de dificultades, que varía entre 0 y 40. Una puntuación total mayor o igual a 20 se considera, según el autor, alterada, entre 16 y 19, límite, y menor o igual a 15, normal, lo que demuestra la ausencia de dificultades psicológicas. La puntuación de la escala de comportamiento prosocial no se incorpora a la puntuación total de dificultades, ya que la ausencia de conductas prosociales es conceptualmente diferente de la presencia de dificultades psicológicas. La interpretación de la puntuación en esta escala difiere porque puntuaciones más altas significan una mayor frecuencia de comportamientos prosociales, a diferencia de la puntuación total, donde valores más altos representan más dificultades. El SDQ lo pueden responder padres, profesores y los propios niños, mayores de 11 años. En esta investigación, los propios adolescentes respondieron las preguntas. 

### Recolección de datos

 En 2018, la recolección de datos se realizó entre junio y agosto mediante formularios creados en *Google Forms* y aplicados durante el horario y en el ambiente escolar de los adolescentes. En 2021, la recolección de datos se realizó entre abril y junio con formularios distribuidos por correo electrónico y *WhatsApp* , debido a la enseñanza remota de emergencia. La secuencia de aplicación de los instrumentos a los estudiantes fue el Formulario de Caracterización de los Participantes, el Cuestionario sobre la Autopercepción de la Salud, el Cuestionario de Calidad de Vida Pediátrica, el Cuestionario de Capacidades y Dificultades (SDQ) y la Escala de Motivación para el Aprendizaje (EMAPRE). 

### Procesamiento y análisis de los datos

Para el análisis de los datos, la información se ingresó cuidadosamente en la base de datos de la investigación y se sometió a tipificación triple para minimizar el riesgo de errores. Se realizaron análisis descriptivo, bivariado y multivariado. El análisis descriptivo consideró la distribución de frecuencia absoluta y relativa de las categóricas (género, edad, año escolar, clasificación económica, autopercepción de la salud, SDQ, calidad de vida, motivación para el aprendizaje) y la síntesis numérica de las continuas (puntaje de la salud). Se realizaron análisis comparativos entre ambos períodos considerando las variables respuesta y explicativas, y para ello se utilizó la prueba de Wilcoxon para las variables continuas, ya que todas tenían una distribución asimétrica, y la prueba de McNemar para las categóricas.

 Para el análisis bivariado se utilizó la prueba Chi-cuadrado de Pearson o la prueba Exacta de Fisher para las variables categóricas, considerando un nivel de significancia del 5%. Inicialmente se consideraron para el análisis multivariado las variables con asociación al nivel de significancia del 20%. Para seleccionar variables en los modelos se adoptó el método manual *backward* , considerando en cada paso del análisis la variable con mayor valor-p para su eliminación del modelo. En el modelo final se mantuvieron las variables con asociación significativa al nivel del 5% y la variable edad, que quedó como variable de ajuste. La magnitud de las asociaciones fue evaluada mediante el *Odds Ratio* (OR) y sus respectivos intervalos de confianza del 95%. Se consideraron estadísticamente significativos los valores de p inferiores al 5% y, para el análisis de los datos, se utilizó el programa estadístico *Statistical Package for the Social Sciences* (SPSS). La adecuación de los modelos se evaluó mediante la prueba de Hosmer y Lemeshow. 

## Resultados

 De los 124 participantes, la mayoría eran mujeres (57,4%) y su edad variaba uniformemente entre 11 y 14 años en la primera etapa, y entre 14 y 17 años en la segunda. La mayoría de los adolescentes (29,4%) se encontraban en el 6º año y el 30,9% en el 9º año, en 2018 y 2021, respectivamente. En cuanto a la clasificación económica, el 58,8% de los adolescentes se encontraban en la clase A ( Figura 2 ). 

En cuanto a la autopercepción de la salud, en 2018 el 91,2% consideraba que tenía buena o excelente salud, y en 2021 este valor se redujo al 80,9%, pero sin una diferencia estadísticamente significativa (p=0,118). El promedio de respuestas sobre el valor atribuido a su salud (cero a 10) se mantuvo estable en ambos periodos en 8,82 (Desviación estándar (DE) =1,12 en 2018 y DE=1,23 en 2021), al igual que la mediana (9,00).

En cuanto a capacidades y dificultades, tanto la puntuación total en la escala SDQ como la de comportamiento prosocial tuvieron una gran mayoría de respuestas “Normal”, y no hubo diferencia significativa en el porcentaje de respuestas entre las categorías en los dos momentos del estudio. En cuanto a la evaluación de la CV, los análisis abarcaron todas sus dimensiones: física, psicosocial (emocional, social y escolar) y general. En la CV evaluada por los padres, hubo una diferencia estadísticamente significativa entre los puntajes de las dimensiones social (p=0,003) y psicosocial (p=0,039), ambas con puntajes más altos en 2021. Entre los adolescentes, hubo diferencias entre los puntajes de CV en el dimensiones física, social, escolar (p=0,006), emocional (p=0,008) y psicosocial (p=0,013). En las variables categóricas la diferencia fue significativa para el puntaje de la dimensión física (p<0,001).

**Figura 2:** - Distribución de las variables sociodemográficas (género, edad y año escolar y clasificación económica) de los adolescentes (n = 68). Belo Horizonte, MG, Brasil, 2018 y 2021

^*^ ES = Educación Secundaria

En el análisis inferencial, al considerar las respuestas de los padres, en 2018, hubo asociación significativa entre la dimensión física y el año escolar (p=0,027) y la clasificación del SDQ total (p=0,056). En 2021, esta dimensión se asoció con la autopercepción de la salud (p=0,031) y la Meta Desempeño-aproximación (p=0,005), y al límite con el género (p=0,058). En la dimensión psicosocial evaluada por los padres hubo asociación significativa en 2018 con la autopercepción de la salud (p=0,035) y al límite con la clasificación del SDQ total (dificultades) (p=0,054), y en 2021 no hubo asociación significativa al nivel del 5 % entre este dominio y las variables probadas. En cuanto a la CV general evaluada por los padres, en 2018 se asoció con la autopercepción de la salud (p=0,021) y la clasificación del SDQ total (p=0,027), y en 2021, con las mismas variables, autopercepción de la salud (p= 0,043) y clasificación del SDQ total (p=0,034).

 Al considerar las respuestas de los adolescentes, en 2018 hubo asociación significativa entre la dimensión física y la clasificación del SDQ total (p=0,003), y en 2021 con la autopercepción de la salud (p=0,001). En la dimensión psicosocial evaluada por los adolescentes, hubo asociación significativa en 2018 con la edad (p=0,036), año escolar (p=0,021) y Meta Aprender (p=0,015), con la clasificación del SDQ total (p) y al límite con la Meta Desempeño-evitación (p=0,054). En 2021, el dominio psicosocial se asoció con el género (p=0,048), al límite con la autopercepción de la salud (p=0,054) y con la clasificación total del SDQ (p=0,009). En cuanto a la CV general evaluada por los adolescentes, en 2018 se asoció con edad (p=0,045), año escolar (p=0,017), Meta Aprender (p=0,028) y clasificación del SDQ total (p). En 2021, la puntuación general de la CV se asoció con la autopercepción de la salud (p=0,026), la Meta Desempeño-evitación (p=0,002) y el SDQ total (p=0,012) (Tablas 1 and 2 ). 

 Para el análisis multivariado se construyeron cuatro modelos. En el primer modelo, la CV se evaluó con base en la percepción de los padres en 2018 y las variables año escolar y SDQ total, asociándose esta última con la CV en el modelo final. Así, el resultado normal del SDQ aumentó 5,37 veces las posibilidades de que el adolescente tenga una calidad de vida buena/excelente en la percepción de los padres. En el segundo modelo, la CV se evaluó a través de la percepción de los adolescentes en 2018 y las variables año escolar, Meta Aprender y Meta Desempeño-evitación. En el modelo final, la calidad de vida se asoció con el año escolar, ya que estar matriculado en el 6º y en el **7º** año aumentó las posibilidades de tener una mejor CV en 4,5 y 9,7 veces, respectivamente, en comparación con los estudiantes del 9º año. En el tercer modelo se evaluó la CV según la percepción de los padres en 2021. Se seleccionaron las variables autopercepción de la salud y SDQ total, siendo esta última asociada a la CV en el modelo final (ajustado por edad). Un resultado normal del SDQ aumentó 5,51 veces las posibilidades de que el adolescente tuviera una calidad de vida buena/excelente en la percepción de sus padres. En el cuarto modelo se evaluó la CV según la percepción de los adolescentes en 2021, seleccionándose las variables género, CCEB, autopercepción de la salud, Meta Desempeño-evitación y SDQ total para ingresar al modelo inicial. Estas dos últimas variables permanecieron asociadas con la CV en el modelo final, de modo que tener un mejor resultado en la escala desempeño-evitación (baja evitación del aprendizaje) aumentó las posibilidades de tener una mejor CV en 5,02 veces, y un resultado normal del SDQ aumentó 11,05 veces las posibilidades de que el adolescente tenga una mejor CV ( Tabla 3 ). 

**Tabla 1:** - Análisis bivariado de la asociación entre las variables del PedsQL ^*^ (padres y adolescentes) y variables seleccionadas (n = 68). Belo Horizonte, MG, Brasil, 2018

**Características**	**Padres** **Dimensiones**						**Adolescentes** **Dimensiones**					
	**Física**		**Psicosocial**		**Calidad de vida general**		**Física**		**Psicosocial**		**Calidad de vida general**	
	alta	baja	alta	baja	alta	baja	alta	baja	alta	baja	alta	baja
	n(%) ^†^	n(%) ^†^	n(%) ^†^	n(%) ^†^	n(%) ^†^	n(%) ^†^	n(%) ^†^	n(%) ^†^	n(%) ^†^	n(%) ^†^	n(%) ^†^	n(%) ^†^
**Género**												
Femenino	19 (55,9)	20 (58,8)	21 (52,5)	18 (64,3)	19 (51,4)	20 (64,5)	24 (66,7)	15 (46,9)	18 (54,5)	21 (60,0)	17 (53,1)	22 (61,1)
Masculino	15 (44,1)	14 (41,2)	19 (47,5)	10 (35,7)	18 (48,6)	11 (35,5)	12 (33,3)	17 (53,1)	15 (45,5)	14 (40,0)	15 (46,9)	14 (38,9)
Valor – ^p‡^	0,806 ^§^		0,333 ^§^		0,274 ^§^		0,100 ^§^		0,649 ^§^		0,506 ^§^	
**Edad (años)**												
11	7 (20,6)	11 (32,4)	10 (25,0)	8 (28,6)	8 (21,6)	10 (32,3)	11 (30,6)	7 (21,9)	7 (21,2)	11 (31,4)	7 (21,9)	11 (30,6)
12	6 (17,6)	8 (23,5)	8 (20,0)	6 (21,4)	8 (21,6)	6 (19,4)	6 (16,7)	8 (25,0)	5 (15,2)	9 (25,7)	5 (15,6)	9 (25,0)
13	8 (23,5)	9 (26,5)	9 (22,5)	8 (28,6)	9 (24,3)	8 (25,8)	7 (19,4)	10 (31,3)	7 (21,2)	10 (28,6)	6 (18,8)	11 (30,6)
14	13 (38,2)	6 (17,6)	13 (32,5)	6 (21,4)	12 (32,5)	7 (22,6)	12 (33,3)	7 (21,9)	14 (42,4)	5 (14,3)	14 (43,8)	5 (13,9)
Valor – p ^‡^	0,077 ^||^		0,494 ^||^		0,309 ^||^		0,932 ^||^		**0,036** ^	|^	**0,045** ^|	^
**Año escolar**												
6º	7 (20,6)	13 (38,2)	10 (25,0)	10 (35,7)	8 (21,6)	12 (38,7)	12 (33,3)	8 (25,0)	8 (24,2)	12 (34,3)	8 (25,0)	12 (33,3)
7º	7 (20,6)	10 (29,4)	10 (25,0)	7 (25,0)	9 (24,3)	8 (25,8)	5 (13,9)	12 (37,4)	5 (15,2)	12 (34,3)	4 (12,5)	13 (36,1)
8º	9 (26,5)	6 (17,6)	10 (25,0)	5 (17,9)	11 (29,7)	4 (12,9)	9 (25,0)	6 (18,8)	8 (24,2)	7 (20,0)	8 (25,0)	7 (19,4)
9º	11 (32,4)	5 (14,7)	10 (25,0)	6 (21,4)	9 (24,3)	7 (22,6)	10 (27,8)	6 (18,8)	12 (36,4)	4 (11,4)	12 (37,5)	4 (11,1)
Valor – p ^‡^	**0,027** ^	|^	0,377 ^||^		0,181 ^||^		0,567 ^||^		**0,021** ^	|^	**0,017** ^|	^
**CCEB** ^¶^												
A1 ^**^	21 (61,8)	19 (55,9)	23 (57,5)	17 (60,7)	21 (56,8)	19 (61,3)	22 (61,1)	18 (56,3)	21 (63,6)	19 (54,3)	20 (62,5)	20 (55,6)
B1/B2 ^††^	13 (38,2)	15 (44,1)	17 (42,5)	11 (39,3)	16 (43,2)	12 (38,7)	14 (38,9)	14 (43,8)	12 (36,4)	16 (45,7)	12 (37,5)	16 (44,4)
Valor – p ^‡^	0,622		0,791 ^§^		0,705 ^§^		0,684 ^§^		0,434 ^§^		0,561 ^§^	
**Autopercepción de la salud**												
Mala/Regular	5 (14,7)	1 (2,9)	6 (15,0)	0 (0,0)	6 (16,2)	0 (0,0)	4 (11,1)	2 (6,3)	4 (12,1)	2 (5,7)	4 (12,5)	2 (5,6)
Buena/Excelente	29 (85,3)	33 (97,1)	34 (85,0)	28 (100,0)	31 (83,8)	31 (100,0)	32 (88,9)	30 (93,7)	29 (87,9)	33 (94,3)	28 (87,5)	34 (94,4)
Valor – p ^‡^	0,099 ^‡‡^		**0,035** ^‡‡^		**0,021** ^‡‡^		0,395 ^‡‡^		0,308 ^‡‡^		0,281 ^‡‡^	
**Meta Aprender**												
< 31	18 (52,9)	17 (50,0)	21 (52,5)	14 (50,0)	20 (54,1)	15 (48,4)	20 (55,6)	15 (46,9)	22 (66,7)	13 (37,1)	21 (65,6)	14 (38,9)
≥ 31	16 (47,1)	17 (50,0)	19 (47,5)	14 (50,0)	17(45,9)	16 (51,6)	16 (44,4)	17 (53,1)	11 (33,3)	22 (66,9)	11 (34,4)	22 (61,1)
Valor – p ^‡^	0,808 ^§^		0,839 ^§^		0,641 ^§^		0,475 ^§^		**0,015** ^§^		**0,028** ^§^	
**Meta Desempeño-aproximación**												
< 15	18 (52,9)	15 (44,1)	20 (50,0)	13 (46,4)	19 (51,4)	14 (45,2)	17 (47,2)	16 (50,0)	15 (45,5)	18 (51,4)	14 (43,7)	19 (52,8)
≥ 15	16 (47,1)	19 (55,9)	20 (50,0)	15 (53,6)	18 (48,6)	17 (54,8)	19 (52,8)	16 (50,0)	18 (54,5)	17 (48,6)	18 (56,3)	17 (47,2)
Valor – p ^‡^	0,467 ^§^		0,772 ^§^		0,611 ^§^		0,819 ^§^		0,622 ^§^		0,457 ^§^	
**Meta Desempeño-evitación**												
≤ 8	19 (55,9)	18 (52,9)	19 (47,5)	18 (64,3)	19 (51,4)	18 (58,1)	19 (52,8)	18 (56,3)	14 (42,4)	23 (65,7)	14 (43,7)	23 (63,9)
> 8	15 (44,1)	16 (47,1)	21 (52,5)	10 (35,7)	18 (48,6)	13 (41,9)	17 (47,2)	14 (43,8)	19 (57,6)	12 (34,3)	18 (56,3)	13 (36,1)
Valor – p ^‡^	0,808 ^§^		0,171 ^§^		0,580 ^§^		0,774 ^§^		**0,054** ^§^		0,096 ^§^	
**SDQ** ^§§^ **Comportamiento Prosocial**												
Normal	33 (97,1)	32 (94,1)	39 (97,5)	26 (92,9)	36 (97,3)	29 (93,5)	34 (94,4)	31 (96,9)	31 (93,9)	34 (97,1)	30 (93,8)	35 (97,2)
Alterado ^||^	1 (2,9)	2 (5,9)	1 (2,5)	2 (7,1)	1 (2,7)	2 (6,5)	2 (5,6)	1 (3,1)	2 (6,1)	1 (2,9)	2 (6,3)	1 (2,8)
Valor – p ^‡^	0,500 ^‡‡^		0,367 ^‡‡^		0,433 ^‡‡^		0,545 ^‡‡^		0,478 ^‡‡^		0,455 ^‡‡^	
**SDQ** ^§§^ **Total**												
Normal	25 (73,5)	31 (91,2)	30 (75,0)	26 (92,9)	27 (73,0)	29 (93,5)	25 (69,4)	31 (96,9)	21 (63,6)	35 (100,0)	20 (62,5)	36 (100,0)
Alterado ^||^	9 (26,5)	3 (8,8)	10 (25,0)	2 (7,1)	10 (27,0)	2 (6,5)	11 (30,6)	1 (3,1)	12 (36,4)	0 (0,0)	12 (37,5)	0 (0,0)
Valor – p ^‡^	**0,056** ^§^		**0,054** ^‡‡^		**0,027** ^§^		**0,003** ^§^		**<0,001** ^§^		**<0,001** ^§^	

^*^ PedsQL = Cuestionario de Calidad de Vida Pediátrica;

^†^ N(%) = Número de participantes y porcentaje de participantes;

^¶^ CCEB = *Critério de Classificação Econômica Brasil* ;

^**^ A1 = Clase A1;

^††^ B1/B2 = Clase B1/B2;

^§§^ SDQ = *The Strengths and Difficulties Questionnaire*

^||^ Asociación lineal por lineal;

^‡^ Valor – p;

^§^ Chi-cuadrado de Pearson;

^‡‡^ Exacta de Fisher;

**Tabla 2:** - Análisis bivariado de la asociación entre las variables del PedsQL ^*^ (padres y adolescentes) y variables seleccionadas (n = 68). Belo Horizonte, MG, Brasil, 2021

**Características**	**Padres** **Dimensiones**						**Adolescentes** **Dimensiones**					
	**Física**		**Psicosocial**		**Calidad de vida general**		**Física**		**Psicosocial**		**Calidad de vida general**	
	alta	baja	alta	baja	alta	baja	alta	baja	alta	baja	alta	baja
	n(%) ^†^	n(%) ^†^	n(%) ^†^	n(%) ^†^	n(%) ^†^	n(%) ^†^	n(%) ^†^	n(%) ^†^	n(%) ^†^	n(%) ^†^	n(%) ^†^	n(%) ^†^
**Género**												
Femenino	30 (65,2)	9 (40,9)	18 (62,1)	21 (53,8)	19 (63,3)	20 (52,6)	12 (75,0)	27 (51,9)	28 (66,7)	11 (42,3)	23 (69,7)	16 (47,1)
Masculino	16 (34,8)	13 (59,1)	11 (37,9)	18 (46,2)	11 36,7)	18 (47,4)	4 (25,0)	25 (48,1)	14 (33,3)	15 (57,7)	10 (30,3)	18 (52,9)
Valor – p ^‡^	0,058 ^§^		0,498 ^§^		0,376 ^§^		0,103 ^§^		0,048 ^§^		0,060 ^§^	
**Edad (años)**												
11	14 (30,4)	6 (27,3)	9 (31,0)	11 (28,2)	9 (30,0)	11 (28,9)	5 (31,3)	15 (28,8)	13 (31,0)	7 (26,9)	12 (36,4)	8 (23,5)
12	9 (19,6)	7 (31,8)	5 (17,2)	11 (28,2)	6 (20,0)	10 (26,3)	2 (12,5)	14 (26,9)	9 (21,4)	7 (26,9)	5 (15,2)	10 (29,5)
13	13 (28,3)	4 (18,2)	6 (20,7)	11 (28,2)	7 (23,3)	10 (26,3)	5 (31,3)	12 (23,1)	11 (26,2)	6 (23,1)	9 (27,3)	8 (23,5)
14	10 (21,7)	5 (22,7)	9 (31,0)	6 (15,4)	8 (26,7)	7 (18,5)	4 (25,0)	11 (21,2)	9 (21,4)	6 (23,1)	7 (21,2)	8 (23,5)
Valor – p ^‡^	0,867 ^||^		0,451 ^||^		0,653 ^||^		0,678 ^||^		0,882 ^||^		0,623 ^||^	
**Año escolar**												
8º	1 (2,2)	0 (0,0)	0 (0,0)	1 (2,6)	1 (3,3)	10 (26,3)	1 (6,3)	0 (0,0)	1 (2,4)	0 (0,0)	1 (3,0)	0 (0,0)
9º	13 (28,3)	8 (36,4)	9 (31,0)	12 (30,8)	9 (30,0)	12 (31,6)	5 (31,3)	16 (30,8)	12 (28,6)	9 (34,6)	11 (33,3)	10 (29,4)
1º año ES ^¶^	11 (23,9)	5 (22,7)	6 (20,7)	10 (25,6)	6 (20,0)	10 (26,3)	1 (6,3)	15 (28,8)	10 (23,8)	6 (23,1)	6 (18,2)	9 (26,5)
2º año ES ^¶^	10 (21,7)	4 (18,2)	5 (17,3)	9 (23,1)	5 (16,7)	9 (23,9)	3 (18,8)	11 (21,2)	8 (19,0)	6 (23,1)	6 (18,2)	8 (23,5)
3º año ES ^¶^	11 (23,9)	5 (22,7)	9 (31,0)	7 (17,9)	9 (30,0)	7 (18,4)	6 (37,5)	10 (19,2)	11 (26,2)	5 (19,2)	9 (27,3)	7 (20,6)
Valor – p ^‡^	0,754 ^||^		0,389 ^||^		0,704 ^||^		−		0,707 ^||^		0,947 ^||^	
**CCEB** ^**^												
A1 ^††^	28 (60,9)	12 (54,5)	17 (58,6)	23 (59,0)	19 (63,3)	21 (55,3)	12 (75,0)	28 (53,8)	27 (64,3)	13 (50,0)	22 (66,7)	17 (50,0)
B1/B2 ^‡‡^	18 (39,1)	10 (45,5)	12 (41,4)	16 (41,0)	11 (36,7)	17 (44,7)	4 (25,0)	24 (46,2)	15 (35,7)	13 (50,0)	11 (33,3)	17 (50,0)
Valor – p ^‡^	0,620 ^§^		0,977 ^§^		0,502 ^§^		0,133		0,245 ^§^		0,167 ^§^	
**Autopercepción de la salud**												
Mala/Regular	12 (26,1)	1 (4,5)	7 (24,1)	6 (15,4)	9 (30,0)	4 (10,5)	8 (50,0)	5 (9,6)	11 (26,2)	2 (7,7)	10 (30,3)	3 (8,8)
Buena/Excelente	34 (73,9)	21 (95,5)	22 (75,9)	33 (84,6)	21 (70,0)	34 (89,5)	8 (50,0)	47 (90,4)	31 (73,8)	24 (92,3)	23 (69,7)	31 (91,2)
Valor – p ^‡^	0,031 ^§§^		0,364 ^§§^		0,043 ^§§^		0,001 ^§§^		0,054 ^§§^		0,026 ^§^	
**Meta Aprender**												
< 31	30 (65,2)	13 (59,1)	20 (69,0)	14 (50,0)	20 (66,7)	23 (60,50)	10 (62,5)	33 (63,5)	28 (66,7)	15 (57,7)	22 (66,7)	20 (58,8)
≥ 31	16 (34,8)	9 (40,9)	9 (31,0)	14 (50,0)	10 (33,3)	15 (39,5)	6 (37,5)	19 (36,5)	14 (33,3)	11 (42,3)	11 (33,3)	14 (41,2)
Valor – p ^‡^	0,624 ^§^		0,398 ^§^		0,602 ^§^		0,944 ^§^		0,456 ^§^		0,507 ^§^	
**Meta Desempeño-aproximación**												
< 15	21 (45,7)	18 (81,8)	17 (58,6)	22 (56,4)	17 (56,7)	22 (57,9)	10 (62,5)	29 (55,8)	23 (54,8)	16 (61,5)	19 (57,6)	20 (58,8)
≥ 15	25 (54,3)	4 (18,2)	12 (41,4)	17 (43,6)	13 (43,3)	16 (42,1)	6 (37,5)	23 (44,2)	19 (45,2)	10 (38,5)	14 (42,4)	14 (47,2)
Valor – p ^‡^	0,005 ^§^		0,855 ^§^		0,919 ^§^		0,634 ^§^		0,583 ^§^		0,918 ^§^	
**Meta Desempeño-evitación**												
≤ 8	22 (47,8)	10 (45,5)	14 (48,3)	18 (46,2)	14 (46,7)	18 (47,4)	4 (25,0)	28 (53,8)	16 (38,1)	16 (61,5)	9 (27,3)	22 (64,7)
> 8	24 (52,2)	12 (54,5)	15 (51,7)	21 (53,8)	16 (53,3)	20 (52,6)	12 (75,0)	24 (46,2)	26 (61,9)	10 (38,5)	24 (72,7)	12 (35,3)
Valor – p ^‡^	0,855 ^§^		0,862 ^§^		0,954 ^§^		0,043 ^§^		0,060 ^§^		0,002 ^§^	
**SDQ** ^||||^ **Comportamiento Prosocial**												
Normal	46 (100,0)	21 (95,5)	29 (100,0)	38 (97,4)	30 (100,0)	37 (97,4)	16 (100,0)	51 (98,1)	41 (97,6)	26 (100,0)	32 (97,0)	34 (100,0)
Alterado ^||^	0 (0,0)	1 (4,5)	0 (0,0)	1 (2,6)	0 (0,0)	1 (2,6)	0 (0,0)	1 (1,9)	1 (2,4)	0 (0,0)	1 (3,0)	0 (0,0)
Valor – p ^‡^	0,324 ^§§^		0,574 ^§^		0,559 ^§§^		0,765 ^§§^		0,618 ^§§^		0,493 ^§§^	
**SDQ* ^||||^ **Total**												
Normal	40 (87,0)	19 (86,4)	23 (79,3)	36 (92,3)	23 (76,7)	36 (94,7)	12 (75,0)	47 (90,4)	33 (78,6)	26 (100,0)	25 (75,8)	33 (97,1)
Alterado ^||^	6 (13,0)	3 (13,6)	6 (20,7)	3 (7,7)	7 (23,3)	2 (5,3)	4 (25,0)	5 (9,6)	9 (21,4)	0 (0,0)	8 (24,2)	1 (0,0)
Valor – p ^‡^	0,610 ^§§^		0,115 ^§§^		0,034 ^§§^		0,124 ^§§^		0,009 ^§§^		0,012 ^§§^	

^*^ PedsQL = Cuestionario de Calidad de Vida Pediátrica;

^†^ N(%) = Número de participantes y porcentaje de participantes;

^¶^ ES = Educación Secundaria;

^**^ CCEB = *Critério de Classificação Econômica Brasil* ;

^††^ A1 = Clase A1;

^‡‡^ B1/B2 = Clase B1/B2;

^§^ Chi-cuadrado de Pearson;

^||||^ SDQ = *The Strengths and Difficulties Questionnaire*

^||^ Asociación lineal por lineal;

^‡^ Valor – p;

^§§^ Exacta de Fisher;

**Tabla 3:** - Resultados de los análisis de regresión logística multivariada entre calidad de vida (PedsQL ^*^ ) según padres y adolescentes (n = 68). Belo Horizonte, MG, Brasil, 2018 y 2021

**Características**		**Modelo 1** ^†^ **- Calidad de Vida General Padres 2018**				**Modelo 2** ^‡^ **- Calidad de Vida General Adolescentes 2018**			
		Modelo inicial		Modelo final		Modelo inicial		Modelo final	
		OR (IC95%) ^§^	Valor-p ^||^	OR (IC95%) ^§^	Valor-p ^||^	OR (IC 95%) ^§^	Valor-p ^¶^	OR (IC 95%) ^§^	Valor-p ^¶^
Año Escolar	6º	1,92 (0,47-7,90)	0,368	−	−	4,31 (0,99-18,78)	0,052	**4,50 (1,06-19,04)**	**0,041**
	7º	0,85 (0,20-3,58)	0,826	−	−	11,06 (2,15-56,72)	0,004	**9,75 (1,98-47,94)**	**0,005**
	8º	0,41 (0,08-1,97)	0,264	−	−	2,28 (0,48-10,76)	0,299	2,65 (0,57-11,99)	0,213
Autopercepción de la salud		−	−	−	−	−	−	−	−
Meta Aprender		−	−	−	−	2,07 (0,66-6,53)	0,212	−	−
Meta Desempeño-Evitación		−	−	−	−	1,61 (0,54-4,82)	0,392		
SDQ ^**^ Comportamiento Prosocial		−	−	−	−	−	−	−	−
SDQ ^**^ (total)		6,21 (1,16-33,16)	0,033	**5,35 (1,06-26,9)**	**0,042**	−	−	−	−
**Características**		**Modelo 3** ^††^ **- Calidad de Vida General Padres 2021**				**Modelo 4** ^‡‡^ **- Calidad de Vida General Adolescentes 2021**			
		Modelo inicial		Modelo final		Modelo inicial		Modelo final	
		OR (IC 95%) ^§^	Valor-p ^§§^	OR (IC 95%) ^§^	Valor-p ^§§^	OR (IC 95%) ^§^	Valor-p ^||||^	OR (IC 95%) ^§^	Valor-p ^||||^
Género		−	−	−	−	1,78 (0,56-5,62)	0,325		
CCEB ^¶¶^						2,26 (0,71-7,19)	0,167	−	−
Autopercepción de la salud		2,93 (0,76-11,29)	0,118	−	−	2,52 (0,51-12,38)	0,256	−	−
Meta Desempeño-Evitación		−	−	−	−	4,44 (1,39-14,11)	0,011	**5,02 (1,67-15,12)**	**0,004**
SDQ ^**^ Comportamiento Prosocial		−	−	−	−	−	−	−	−
SDQ ^**^ (total)		4,36 (0,79-23,96)	0,090	**5,51 (1,05-28,98)**	**0,044**	7,83 (0,78-78,58)	0,080	**11,05 (1,18-103,77)**	**0,035**

^*^ PedsQL = Cuestionario de Calidad de Vida Pediátrica;

^†^ Modelo 1 = 9º año, SDQ Total: Alterado, Autopercepción de la salud mala/regular no entró en el modelo porque no convergió debido a la categoría sin observaciones, Modelo Final ajustado por edad;

^‡^ Modelo 2 = 9º año, Autopercepción de la salud mala/regular, Meta Aprender y Meta Desempeño-evitación: puntuación más alta, SDQ no entró en el modelo porque no convergió debido a la categoría sin observaciones;

^||^ Test de Wald, Ajuste de los modelos inicial/final (Hosmer y Lemeshow): p=0,638/p=0,576;

^¶^ Prueba de Wald, Ajuste de los modelos inicial/final (Hosmer y Lemeshow): p=0,639/p=1,000;

^††^ Modelo 3 = Autopercepción de la salud mala/regular, SDQ Total: Alterado, Modelo Final ajustado por edad;

^‡‡^ Modelo 4 = Género masculino, Autopercepción de la salud mala/regular, Meta Desempeño-evitación: puntuación más alta, SDQ Total: Alterado;

^§^ OR(IC95%) = *Odds Ratio* e intervalo de confianza del 95%;

^§§^ Prueba de Wald, Ajuste de los modelos inicial/final (Hosmer y Lemeshow): p=0,713/p=0,834;

^||||^ Prueba de Wald, Ajuste de los modelos inicial/final (Hosmer y Lemeshow): p=0,700/p=0,289;

^¶¶^ CCEB = *Critério de Classificação Econômica Brasil*

^**^ SDQ = *The Strengths and Difficulties Questionnaire* ;

En resumen, considerando los análisis de regresión logística en ambos períodos, la variable Capacidades y Dificultades, medida por el puntaje total del SDQ, probada en los modelos 1 y 3, se asoció con la calidad de vida en la evaluación de los padres en 2018 y 2021. Según la percepción de los adolescentes, la calidad de vida estuvo asociada, en 2018, al año escolar y en 2021, a la Meta Desempeño-evitación y al SDQ total.

## Discusión

 La salud y los determinantes sociales de la salud encuentran, en el ámbito escolar, un campo de estudio y reflexión, ya que la edad escolar es un período crucial de crecimiento físico y cognitivo ^(^ ^27^ ^)^ . Como el contexto de la investigación era una institución de financiamiento privado, los participantes pertenecían a estratos más altos en términos de clase económica. Al comparar los períodos 2018 y 2021, se ha producido un empeoramiento de la valoración de la autopercepción de la salud, ya que el 8,8% y el 19,2%, respectivamente, consideraron su salud regular, mala o muy mala, pesando la situación de emergencia en salud pública. La suspensión de clases presenciales y la adaptación de la rutina y los ciclos escolares al contexto remoto provocaron inseguridad e incertidumbre, y el aislamiento social, con reducido contacto con los pares, afectó el bienestar de los adolescentes ^(^ ^17^ ^-^ ^18^ ^)^ . La escuela es un espacio colectivo favorable a la promoción de la salud, que ofrece a niños y adolescentes un campo para concienciar y practicar hábitos de vida saludables, autocuidado y prevención de riesgos ^(^ ^4^ ^,^ ^15^ ^)^ . 

 Siendo la adolescencia una etapa crítica y sensible en la vida de las personas y sus familias, en la que se experimentan profundas transformaciones físicas, emocionales ^(^ ^28^ ^)^ , sociales y cognitivas, es importante monitorear la salud y el bienestar de diferentes aspectos, siendo la calidad de vida un indicador muy apropiado ^(^ ^29^ ^)^ . En cuanto a la valoración de la calidad de vida en la percepción de los padres, el aumento en el puntaje de la dimensión psicosocial (emocional, social y escolar) evidenciado por el estudio puede explicarse por el uso de recursos tecnológicos accesibles al estrato social estudiado, y la disminución en la puntuación de la dimensión física refleja la crisis sanitaria y epidemiológica ocurrida a raíz de la propagación del SARS-CoV-2. Además de los daños en el ámbito educativo, el cierre de las escuelas generó un cambio significativo en la dinámica de las familias, que necesitaban conciliar los compromisos profesionales de los padres con las demandas estudiantiles de los adolescentes en espacios más amplios de tiempo compartido ^(^ ^30^ ^)^ , lo que implicó una mayor interacción entre los padres en la vida escolar de los adolescentes ^(^ ^14^ ^)^ . Al observar la calidad de vida a partir del autorreporte de los adolescentes, la disminución de los puntajes en la dimensión psicosocial puede justificarse por la alta demanda de interacción con pares inherente a la fase de la adolescencia, que fue obstaculizada por las necesarias medidas de distanciamiento social. Se sabe que la interacción y la comunicación son necesidades naturales del ser humano ^(^ ^10^ ^)^ , y el ambiente escolar es un lugar de crecimiento, desarrollo de habilidades cognitivas y conductuales y socialización con repercusiones en la calidad de vida de niños y adolescentes ^(^ ^8^ ^,^ ^31^ ^)^ . 

 Además, vinculados a la calidad de vida, aspectos como el género, la educación del padre y de la madre y los ingresos familiares fueron investigados en un estudio realizado con adolescentes iraníes de entre 15 y 18 años, que mostró efectos significativos de la desigualdad socioeconómica en la calidad de vida relacionada con la salud de los adolescentes iraníes, lo que indica la necesidad de políticas públicas a favor de una sociedad sana para una mejor calidad de vida ^(^ ^29^ ^)^ . 

 En el análisis de la motivación para aprender, en la comparación entre los períodos estudiados, se evidenció una caída en la Meta Aprender y un aumento en la Meta Desempeño-evitación, condiciones determinadas, posiblemente, por razones pedagógicas, con la implementación repentina de un modelo remoto de enseñanza. Adaptar el sistema educativo, involucrando a profesores y estudiantes en torno a contenidos, métodos de enseñanza, de aprendizaje y de evaluación, es uno de los desafíos de este momento histórico, ya que la motivación de los estudiantes para aprender involucra también cuestiones docentes, estructurales y sociales ^(^ ^32^ ^)^ . En cuanto a la inserción de tecnologías de la información y la comunicación, una investigación entre estudiantes de Educación Secundaria brasileños reveló que el 20% de los participantes negaba la suficiencia de las clases remotas. El motivo principal fue la dificultad en la comprensión de los contenidos transmitidos, resultado que hace pensar en la interacción remota entre docente y alumno y lo motivadora y efectiva que puede resultar para la educación básica ^(^ ^33^ ^)^ . 

 La interrupción de la rutina escolar presencial también repercutió en el interés de los adolescentes de una institución pública de la región sureste de Brasil -región que no presenta los mayores porcentajes de exclusión escolar del país-, donde casi el 40% de los estudiantes indicaron falta de interés en las actividades escolares a distancia y otros 49% manifestaron tendencia a la deserción escolar ^(^ ^2^ ^)^ . Respecto al segmento de los últimos años de la Educación Primaria, los resultados de otra investigación realizada durante el período de pandemia mostraron que los estudiantes que habitualmente estaban motivados intrínsecamente para aprender, estaban motivados extrínsecamente o desmotivados con respecto a la enseñanza remota, sugiriendo implicaciones directas entre la calidad del compromiso entre profesor y alumno y la motivación para aprender en esta modalidad de enseñanza ^(^ ^34^ ^)^ . Para los estudiantes universitarios, la reanudación de las actividades presenciales representó una mejora en los niveles de motivación para estudiar, permeado por el contacto social ^(^ ^35^ ^)^ . 

 En este sentido, los valores de razón de oportunidades encontrados en los análisis de regresión logística multivariada realizados con la muestra del presente estudio arrojaron resultados interesantes. Las mayores posibilidades de tener una mejor calidad de vida autorreportada encontrada entre los adolescentes de los primeros años de la Educación Primaria II corroboraron estudios prepandémicos ^(^ ^36^ ^-^ ^37^ ^)^ , que demostraron que los estudiantes más jóvenes tienen una mejor calidad de vida general en comparación con los estudiantes en los últimos años en diferentes estratos educativos. Una investigación longitudinal realizada en 2018 con estudiantes adolescentes chinos mostró una caída en la satisfacción con la vida y un aumento paulatino de la desesperanza en la carrera académica, asociados a aspectos individuales y contextuales, como la resiliencia, los factores socioculturales y el entorno familiar ^(^ ^38^ ^)^ . 

 En ambos períodos investigados, el comportamiento típico del adolescente le favoreció a tener una buena o excelente calidad de vida en la percepción de sus padres y en su propia valoración, donde tener un resultado normal en el análisis de capacidades y dificultades, relacionado con la salud mental del estudiante, aumentó más de cinco veces las posibilidades de tener una mejor calidad de vida. Este dato sugiere un acercamiento entre los constructos de salud mental y calidad de vida. La acción conjunta de los profesionales de la salud y la educación en la escuela contribuye a la implementación y desarrollo de acciones para los adolescentes en busca de comportamientos saludables hacia ellos mismos, sus actividades y el grupo del que forman parte ^(^ ^3^ ^,^ ^39^ ^-^ ^40^ ^)^ . 

 Así, también es importante resaltar el vínculo entre la salud física y psicosocial y el comportamiento motivacional adoptado por el adolescente hacia el aprendizaje, ya que los estudiantes que presentaron resultado adecuado en el análisis de capacidades y dificultades y una mejor calidad de motivación para aprender, indicada por una baja evitación del aprendizaje, tenían entre cinco y once veces más probabilidades de tener una mejor calidad de vida general en el análisis del período pandémico. La salud, en sus múltiples dimensiones, dialoga con una conducta adecuada en el ámbito escolar, incluido el cumplimiento de las tareas académicas. La presencia de factores de riesgo para problemas de salud mental, por ejemplo, afectó a la calidad de vida relacionada con la salud de adolescentes alemanes, como muestra un estudio longitudinal de 2017 ^(^ ^41^ ^)^ , en consonancia con el impacto negativo sobre la calidad de vida observado en los jóvenes universitarios brasileños con síntomas de depresión, ansiedad y estrés ^(^ ^42^ ^)^ . 

 Las condiciones psicológicas y los trastornos emocionales no son raros entre los adolescentes ^(^ ^43^ ^)^ , y la pandemia causada por el SARS-CoV-2 contribuyó a la epidemiología de estos trastornos. Según estudios chinos realizados en 2020 con adolescentes de 12 a 18 años y jóvenes, la prevalencia de síntomas depresivos, de ansiedad, estrés postraumático y otros problemas psicológicos es alta. El acceso a información sobre la enfermedad y las medidas de prevención fue considerado un factor protector contra el desarrollo de síntomas emocionales ^(^ ^19^ ^-^ ^20^ ^)^ . Asimismo, investigaciones realizadas con niños y adolescentes en Alemania en 2020 ^(^ ^21^ ^)^ y en Brasil en 2022 ^(^ ^18^ ^)^ indicaron que dos tercios de los participantes informaron estar muy abrumados y que los adolescentes expresaron mayores sentimientos de soledad, ansiedad y tristeza debido a la pandemia. La calidad de vida relacionada con la salud autorreportada fue significativamente menor en comparación con el período anterior a la crisis sanitaria, además de reportar más problemas de salud mental y mayores niveles de ansiedad. Además, los niños y adolescentes de menor nivel socioeconómico fueron más impactados, reforzando la necesidad de implementar estrategias de promoción de la salud y prevención de enfermedades para mantener la salud mental y mejorar la calidad de vida de los niños y adolescentes, especialmente aquellos que se encuentran en una situación de mayor vulnerabilidad ^(^ ^44^ ^)^ . 

El presente estudio destacó las repercusiones pedagógicas, formativas, de salud física y socioemocional de adolescentes que cursaban los últimos años de la Educación Primaria durante el período de la pandemia provocada por el SARS-CoV-2. La pérdida de seguimiento de parte del grupo investigado en 2018 constituyó una limitación de la investigación. Al tratarse de una muestra de un único estrato social, estudiantes de una institución de financiamiento privado, los resultados no representan la realidad masiva brasileña, y es deseable realizar más investigaciones para ampliar las conclusiones.

Los aportes de este trabajo al avance del conocimiento científico en el área de la salud abarcan una gama de factores, intrínsecos y extrínsecos al individuo, inherentes al desarrollo de la infancia y la adolescencia, en los que la acción intersectorial entre los servicios de salud y las instituciones educativas puede favorecer a la detección precoz de enfermedades, promoviendo la salud en su sentido más amplio y constituyendo un campo de actividad significativo para los profesionales de Enfermería.

## Conclusión

La mayoría de los participantes eran mujeres y pertenecían a la clase A. En la comparación entre 2018 y 2021, se produjo un empeoramiento en la evaluación de la autopercepción de la salud. El promedio de las respuestas sobre el valor atribuido a la salud se mantuvo estable en ambos períodos. En la evaluación de la CV por parte de los padres, hubo diferencia estadísticamente significativa entre los puntajes de las dimensiones social y psicosocial, ambas con puntajes más altos en 2021. Entre los adolescentes, hubo diferencias entre los puntajes de la CV en las dimensiones física, social, escolar, emocional y psicosocial. Para las capacidades y dificultades, no hubo diferencia significativa en el porcentaje de respuestas entre las categorías en las dos etapas del estudio. El adolescente con puntaje normal en la evaluación de la salud mental tuvo mayores posibilidades de tener una mejor calidad de vida durante los períodos investigados (OR=5,35 y OR=5,51). Los estudiantes de los primeros años de Educación Primaria II mostraron más motivación para aprender, aumentando la posibilidad de mejorar la calidad de vida autorreportada en los dos periodos, respectivamente, hasta 9,7 y 5,02 veces.

En resumen, la asociación entre salud, calidad de vida, comportamiento y motivación para aprender entre estudiantes adolescentes suscita importantes reflexiones sobre los abordajes de la salud adolescente en el ambiente escolar. Se destaca la urgencia de una mirada más amplia a este espacio, que debe cumplir importantes funciones más allá de las educativas curriculares, atribuyendo significados y construyendo una educación cada vez más eficaz e integral.

# Translation: Qualidade de vida, percepção de saúde, motivação para aprendizagem e comportamento de adolescentes em instituição de ensino ^*^

**DOI:** 10.1590/1518-8345.6919.4339

**Authors:** Graziela Nunes Alfenas Fernandes, Stela Maris Aguiar Lemos

**Affiliations:** ^1^ Universidade Federal de Minas Gerais, Faculdade de Medicina, Belo Horizonte, MG, Brasil.; ^2^ Bolsista da Coordenação de Aperfeiçoamento de Pessoal de Nível Superior (CAPES), Brasil.

**Keywords:** Adolescente, Saúde, Qualidade de Vida, Comportamento, Ensino Remoto, Percepção

## Abstract

**Objetivo:**

analisar as variáveis sociodemográficas, qualidade de vida, autopercepção de saúde, motivação para aprendizagem e comportamento de adolescentes em 2018 e 2021.

**Método:**

estudo transversal observacional com 124 adolescentes em 2018 e 68 em 2021. Utilizou-se para a coleta de dados um Formulário para as variáveis sociodemográficas, Questionário Pediátrico de Qualidade de Vida, instrumento de Autopercepção de Saúde, Escala de Motivação para a Aprendizagem e Questionário de Capacidades e Dificuldades. A coleta dos dados foi realizada por formulários no *Google Forms* . Para a análise, utilizou-se estatística descritiva e regressão logística.

**Resultados:**

a maioria dos participantes pertencia à classe A. Na comparação entre 2018 e 2021, houve piora da avaliação de autopercepção de saúde. Na avaliação da QV pelos pais, houve diferença estatística significativa entre os escores das dimensões social e psicossocial. Entre os adolescentes, houve diferenças entre os escores da QV nas dimensões física e psicossocial.

**Conclusão:**

o adolescente com melhor avaliação da saúde mental teve maior chance de ter melhor qualidade de vida nos períodos investigados (OR=5,35 e OR=5,51). Estudantes mais novos apresentaram maior motivação para aprender, sendo que aumentaram em até 9,75 e 5,02 vezes a chance de melhoria da qualidade de vida nos dois períodos, respectivamente.

## Destaques

**(1)** O ambiente escolar é um espaço favorável à promoção da saúde física, mental e social.

**(2)** A conscientização sobre hábitos de vida saudáveis deve ser feita na adolescência.

**(3)** Aspectos relacionados com a saúde podem influenciar o comportamento e a aprendizagem.

**(4)** A qualidade de vida (QV) e suas dimensões relacionam-se à motivação para aprender.

**(5)** A saúde mental do adolescente está relacionada com a autopercepção da QV.

## Introdução

 Uma importante crise em Saúde Pública trouxe mudanças globais significativas entre os anos 2019 e 2021 no que se refere à maneira como as pessoas vivem e convivem, criando a necessidade imediata de adaptações de toda ordem. A pandemia causada pelo coronavírus 2 da Síndrome Respiratória Aguda Grave (SARS-CoV-2) mudou a forma de pensar e praticar a educação no Brasil e no mundo, afetando mais de 90% dos estudantes de todo o planeta e os processos educacionais em mais de 180 países. No Brasil, medidas de distanciamento social para mitigação do vírus acarretaram a suspensão das atividades escolares presenciais e decretos governamentais autorizaram o Ensino Remoto Emergencial (ERE), implementando, subitamente, atividades escolares por meio de tecnologias de informação e comunicação ^(^ ^1^ ^-^ ^3^ ^)^ . 

 O indivíduo, durante a infância e a adolescência, encontra na escola uma dimensão importante de sua qualidade de vida, pois neste espaço, além de adquirir conhecimentos curriculares, desenvolve valores fundamentais para a sua formação integral como ser humano e cidadão. Neste sentido, o ambiente escolar é propício para a realização de programas de promoção e educação em saúde efetivos e abrangentes ^(^ ^4^ ^)^ , na medida em que, por meio da intersetorialidade ^(^ ^5^ ^-^ ^6^ ^)^ , coopera para que construam conhecimentos sobre a natureza humana e seu desenvolvimento, aprimorando habilidades e criando aptidões para o autocuidado, cuidado com o ambiente e bem estar geral ^(^ ^7^ ^-^ ^9^ ^)^ , e reúne demandas cognitivas, físicas, emocionais, familiares e socioeconômicas dos estudantes na direção na saúde integral. 

 Assim, destacada a escola como lugar de cuidado, estímulos e crescimento nos diversos âmbitos da vida, o novo formato de construção do conhecimento trouxe reflexões sobre as características fundamentais do processo didático-pedagógico, envolvendo equipes técnica, docente e discente em torno do ensinar e do aprender ^(^ ^10^ ^)^ . Sabe-se que a motivação humana por realizar tarefas obedece a estímulos internos e a aspectos contextuais. Durante a adolescência, um período caracterizado por transições, a motivação é fundamental para viabilizar que os jovens alcancem seus objetivos e vivenciem um desenvolvimento saudável, incluindo necessidades psicológicas, autodisciplina e incentivo acadêmico ^(^ ^11^ ^)^ . No âmbito da aprendizagem, a motivação pode estar voltada majoritariamente para o aprimoramento individual ou visando, principalmente, a *performance* no cumprimento das atividades acadêmicas ^(^ ^12^ ^)^ . Aliados à motivação para aprender, fatores relacionados à saúde mental ^(^ ^13^ ^)^ , comportamento, aspectos familiares ^(^ ^14^ ^)^ e questões socioeconômicas e demográficas ^(^ ^15^ ^)^ podem influenciar o processo educacional de adolescentes ^(^ ^2^ ^)^ . 

 Estudos realizados no Brasil ^(^ ^16^ ^-^ ^18^ ^)^ e em países como China e Alemanha ^(^ ^19^ ^-^ ^21^ ^)^ evidenciaram piora da saúde mental, autoestima e bem-estar psicossocial dos adolescentes na vigência da pandemia. Os efeitos negativos repercutiram no desenvolvimento e desempenho nos diversos campos da vida. Cabe destacar, ainda, as consequências destes fatores para a qualidade de vida geral e suas dimensões física, emocional, escolar e social nesta população, sublinhadas pela implementação das novas estratégias pedagógicas e didáticas inerentes ao emergencial modelo de educação. 

Desta forma, pensando na educação básica e resgatados o ensino e a aprendizagem como processos de interação que perpassam a vinculação, o desenvolvimento biopsicossocial e a escola, o principal objetivo deste estudo foi analisar as variáveis sociodemográficas, qualidade de vida, autopercepção de saúde, motivação para aprendizagem e o comportamento de adolescentes de uma instituição privada antes da pandemia de COVID-19, em 2018 e 2021.

## Método

### Tipo de estudo e definição da amostra

Trata-se de estudo quantitativo transversal observacional com amostra estratificada por sexo, idade e ano escolar. A amostra foi composta por 124 adolescentes matriculados no Ensino Fundamental II de uma escola brasileira de financiamento privado antes da pandemia de COVID-19 em 2018, e 68 estudantes durante a pandemia de COVID-19 em 2021, onde estavam matriculados aproximadamente 244 estudantes no segmento educacional pesquisado.

 O cálculo amostral considerou 5% de erro amostral, intervalo de 95% de confiança e 15% de prevalência considerando a qualidade de vida como desfecho de interesse, sendo estimada uma amostra de 114 adolescentes selecionados por amostragem aleatória estratificada por sexo e ano escolar. Utilizou-se o teste para estimativa de uma proporção no *software* *Minitab 14 Release* . 

 Para motivação para aprendizagem, um tamanho amostral de 124 indivíduos obteve 80% de poder estatístico na estimativa da baixa motivação para aprender, considerando 22,5% como sendo o parâmetro na população. A precisão que se obtém a partir desse tamanho amostral e poder estatístico é de 10% e o nível de significância foi de 5%. Utilizou-se o teste para estimativa de uma proporção no *software* *Minitab 17* . 

### Critérios de seleção

 Como critérios de inclusão, em 2018 foram considerados os adolescentes matriculados no segmento Ensino Fundamental II na instituição pesquisada, com idade entre 11 e 14 anos e estudantes do 6º ao 9º ano escolar, sendo incluídos 124 adolescentes. Foi utilizado como critério de exclusão o preenchimento incompleto dos instrumentos da pesquisa. Em 2021, os 124 participantes incluídos em 2018 foram convidados a participar da segunda etapa do estudo, 56 se recusaram por motivos como mudança de escola, falta de disponibilidade de tempo para preenchimento dos instrumentos da pesquisa e mudança de contato telefônico, e 68 concordaram com a nova coleta de dados ( Figura 1 ). 

**Figura 1:** - Fluxograma dos participantes da pesquisa 2018-2021

^*^ EFII = Ensino Fundamental II; ^†^ EM = Ensino Médio

### Aspectos éticos

Esta pesquisa foi aprovada pelo Comitê de Ética em Pesquisa da Universidade Federal de Minas Gerais (UFMG) sob Certificado de Apresentação de Apreciação Ética (CAAE) número 80162417.1.0000.5149. O projeto foi aprovado sob número de parecer 2.422.795 em dezembro de 2017 e a emenda do projeto foi aprovada sob número do parecer 4.446.496 em dezembro de 2020. Em 2018, os pais ou responsáveis e os adolescentes participantes assinaram o Termo de Consentimento Livre e Esclarecido (TCLE) e o Termo de Assentimento Livre e Esclarecido (TALE), respectivamente. Em 2021, em razão do ensino remoto emergencial, os participantes aceitaram os TCLE e TALE por meio de formulários eletrônicos.

### Variáveis do estudo

 Para este estudo foram consideradas as variáveis sociodemográficas - sexo, idade, ano escolar e classificação econômica (CCEB); autopercepção de saúde e capacidades e dificuldades (SDQ – Por) como variáveis explicativas, e qualidade de vida, autorrelato e relato dos pais nas dimensões física, psicossocial e qualidade de vida geral (PedsQL ^TM^ ), e motivação para aprender, considerados seus três domínios (EMAPRE), como variáveis resposta. 

### Instrumentos utilizados para a coleta das informações

 Neste estudo foram utilizados cinco instrumentos: questionário contendo as variáveis sociodemográficas dos participantes, o Questionário Pediátrico sobre Qualidade de Vida – PedsQL™ 4.0, o questionário sobre a Autopercepção de Saúde, a Escala de Motivação para a Aprendizagem – EMAPRE e o *The Strengths and Difficulties Questionnaire* – SDQ. 

 Formulário para obtenção das variáveis sociodemográficas (sexo, idade e ano escolar e classificação econômica). Para a classificação da classe econômica, adotou-se o Critério de Classificação Econômica Brasil - CCEB ^(^ ^22^ ^)^ . Para tanto, foi solicitado aos pais e/ou responsáveis pelos adolescentes o preenchimento dos itens referentes ao poder aquisitivo e ao grau de instrução do chefe da família, apenas em 2018, a fim de agrupar posteriormente os participantes nas classes de A a E. 

 Questionário Pediátrico sobre Qualidade de Vida - PedsQL™ 4.0. Esse instrumento foi padronizado e validado para o português do Brasil ^(^ ^23^ ^-^ ^24^ ^)^ , e permite avaliar a qualidade de vida (QV) em quatro domínios: físico (dimensão física), emocional, social e escolar (dimensão psicossocial). Para a sua utilização foi solicitada a permissão de uso aos autores. Além da versão destinada para o preenchimento dos pais, este questionário também tem a sua versão destinada aos adolescentes, para autorrelato da qualidade de vida, e ambas foram utilizadas na pesquisa. Para a obtenção do escore, os itens são invertidos e linearmente transformados em escala de 0 a 100: 0 - 100; 1 - 75; 2 - 50; 3 - 25; 4 - 0. Em seguida, os escores de cada item são somados e divididos pelo número de escores respondidos, apresentando a média de cada item do domínio. Para obtenção dos dados da Dimensão Psicossocial, é realizada a soma dos escores das escalas Emocional, Social e Escolar. Para a Dimensão Física, são utilizadas as respostas obtidas na escala física. Para o escore geral, o cálculo é realizado dividindo a soma de todos os itens pelo número de itens respondidos em todas as escalas. 

Questionário sobre a Autopercepção de Saúde. Esse questionário foi elaborado pelas pesquisadoras com as seguintes questões: “Como você avalia/considera sua saúde atualmente?” e “Que nota você daria para a sua saúde?”. As respostas à primeira questão são avaliadas por meio da escala Likert com as opções: muito ruim, ruim, regular, boa e excelente. Para a segunda questão foi utilizada a escala numérica de 0 a 10 para atribuir a resposta, sendo considerado zero, muito ruim, e 10 excelente.

 Escala de Motivação para a Aprendizagem – EMAPRE ^(^ ^12^ ^)^ . Esse instrumento permite avaliar a motivação dos estudantes para o estudo e a realização das atividades acadêmicas. A escala é composta por 28 questões distribuídas em três domínios: Meta Aprender, Meta Performance-aproximação e Meta Performance-evitação. A Meta Aprender é aquela em que o estudante busca desafios e os utiliza como recurso para a própria aprendizagem e desenvolvimento intelectual. A Meta Performance-aproximação evidencia a preocupação do estudante principalmente em superar os demais demonstrando a própria capacidade. E a Meta Performance-evitação refere-se à condição na qual o estudante evita situações em que pode ocorrer o fracasso na realização das tarefas escolares propostas. Assim, todas as questões se relacionam à motivação, atitude e objetivos com relação à aprendizagem. A EMAPRE é uma escala tipo Likert, com opções de resposta “concordo”, “não sei” e “discordo”. Esse instrumento foi construído com estudantes do Ensino Médio e submetido à análise fatorial na qual os três fatores explicaram 40,56% da variância total na análise dos componentes principais, e os três domínios apresentaram Alpha de Cronbach α=0,795, α=0,798 e α=0,801, respectivamente, na análise da consistência interna, evidenciando a adequação do instrumento para a amostra de adolescentes proposta no estudo ^(^ ^25^ ^)^ . 

 Questionário de Capacidades e Dificuldades (SDQ) ^(^ ^26^ ^)^ . Esse instrumento foi construído em 1997 por Goodman e validado no Brasil por Fleitlich, Cortázar e Goodman em 2000. O SDQ é um questionário que permite rastrear problemas de saúde mental infanto-juvenil. O questionário é composto de 25 itens, distribuídos em cinco escalas, a saber: sintomas emocionais (cinco itens); problemas de comportamento (cinco itens); hiperatividade/desatenção (cinco itens); problemas de relacionamento (cinco itens) e comportamento pró-social (cinco itens). Desses 25 itens, 10 são sobre capacidades, 14 sobre dificuldades e um deles é considerado neutro. Cada um dos itens pode ser respondido como “falso”, “mais ou menos verdadeiro” ou “verdadeiro”. A pontuação para cada uma das escalas é obtida somando-se as pontuações dos 5 itens, gerando uma pontuação que varia de 0 a 10. As pontuações nas escalas de hiperatividade, sintomas emocionais, problemas de conduta e problemas de relacionamento com os pares são somadas para gerarem uma pontuação total de dificuldades, que varia entre 0 a 40. Pontuação total maior ou igual a 20 é considerada, segundo o autor, como alterada, entre 16 a 19, limítrofe, e menor ou igual a 15, normal, o que evidencia ausência de dificuldades psicológicas. A pontuação da escala sobre comportamento pró-social não se incorpora à pontuação total de dificuldades, já que a ausência de comportamentos pró-sociais é conceitualmente diferente da presença de dificuldades psicológicas. A interpretação da pontuação nessa escala se diferencia porque escores mais altos significam maior frequência de comportamentos pró-sociais, ao contrário do escore total, onde valores mais altos representam mais dificuldades. O SDQ pode ser respondido por pais, professores e pelas próprias crianças, acima de 11 anos de idade. Nessa pesquisa, os próprios adolescentes responderam às perguntas. 

### Coleta de dados

 Em 2018, a coleta dos dados foi realizada entre junho e agosto por meio de formulários criados no *Google Forms* e aplicados no horário e no ambiente escolar dos adolescentes. Em 2021, a coleta de dados ocorreu no período entre abril e junho com distribuição dos formulários via *e-mail* e *WhatsApp* , em função do ensino remoto emergencial. A sequência de aplicação dos instrumentos para os estudantes foi o Formulário de Caracterização dos Participantes, o Questionário sobre a Autopercepção de Saúde, o Questionário Pediátrico sobre Qualidade de Vida, o Questionário de Capacidades e Dificuldades (SDQ) e a Escala de Motivação para a Aprendizagem (EMAPRE). 

### Tratamento e análise dos dados

Para a análise dos dados, as informações foram cuidadosamente inseridas no banco de dados da pesquisa e submetidas à digitação tripla, a fim de minimizar o risco de erros. Foram realizadas análises descritiva, bivariada e multivariada. A análise descritiva considerou a distribuição de frequência absoluta e relativa das variáveis categóricas (sexo, idade, ano escolar, classificação econômica, autopercepção de saúde, SDQ, qualidade de vida, motivação para aprendizagem) e de síntese numérica das variáveis contínuas (nota da saúde). As análises comparativas entre os dois períodos foram feitas considerando as variáveis resposta e explicativas e, para tal, utilizou-se o teste de Wilcoxon para as variáveis contínuas, pois todas apresentaram distribuição assimétrica, e o teste de McNemar para as variáveis categóricas.

 Para a análise bivariada, utilizou-se os testes Qui-quadrado de Pearson ou Exato de Fisher para as variáveis categóricas, considerando-se o nível de significância de 5%. As variáveis com associação ao nível de 20% de significância foram, inicialmente, consideradas para a análise multivariada. Para a seleção das variáveis nos modelos, foi adotado o método manual *backward* , considerando em cada passo da análise a variável com maior valor-p para retirada do modelo. No modelo final foram mantidas as variáveis com associação significante ao nível de 5% e a variável idade, que permaneceu como variável de ajuste. A magnitude das associações foi avaliada pelas *Odds Ratio* (OR) e seus respectivos intervalos de confiança a 95%. Valores de p inferiores a 5% foram considerados estatisticamente significantes e, para a análise dos dados, utilizou-se o programa estatístico *Statistical Package for the Social Sciences* (SPSS). A adequação dos modelos foi avaliada pelo teste de Hosmer e Lemeshow. 

## Resultados

 Dos 124 participantes, a maioria dos participantes era do sexo feminino (57,4%) e a idade variou de forma uniforme entre 11 e 14 anos na primeira etapa, e entre 14 e 17 anos na segunda. A maior parte dos adolescentes (29,4%) estava no 6º ano e 30,9% no 9º ano, em 2018 e 2021, respectivamente. Quanto à classificação econômica, 58,8% dos adolescentes na classe A ( Figura 2 ). 

Quanto à autopercepção de saúde, em 2018, 91,2% consideraram que tinham saúde boa ou excelente, e em 2021 esse valor se reduziu para 80,9%, porém sem diferença estatisticamente significante (p=0,118). A média das respostas sobre o valor atribuído à sua saúde (zero a 10) ficou estável nos dois períodos em 8,82 (Desvio padrão (DP) =1,12 em 2018 e DP=1,23 em 2021), assim como a mediana (9,00).

Quanto às capacidades e dificuldades, tanto o escore total da escala SDQ quanto o do comportamento pró-social tiveram uma larga maioria de respostas “Normal”, e não houve diferença significativa no percentual de respostas entre as categorias nos dois momentos do estudo. No que se refere à avaliação da QV, as análises contemplaram todas as suas dimensões: a física, a psicossocial (emocional, social e escolar) e a geral. Na QV avaliada pelos pais, houve diferença estatística significante entre os escores das dimensões social (p=0,003) e psicossocial (p=0,039), ambas com maiores escores em 2021. Entre os adolescentes, houve diferenças entre os escores da QV nas dimensões física, social, escolar (p=0,006), emocional (p=0,008) e psicossocial (p=0,013). Nas variáveis categóricas, a diferença foi significativa para o escore da dimensão física (p<0,001).

**Figura 2:** - Distribuição das variáveis sociodemográficas (sexo, idade e ano escolar e classificação econômica) dos adolescentes (n = 68). Belo Horizonte, MG, Brasil, 2018 e 2021

^*^ EM = Ensino Médio

Na análise inferencial, ao considerar as respostas dos pais, em 2018, houve associação significativa da dimensão física com o ano escolar (p=0,027) e a classificação do SDQ total (p=0,056). Em 2021, essa dimensão foi associada à autopercepção de saúde (p=0,031) e à Meta Performance-aproximação (p=0,005), e limítrofe com o sexo (p=0,058). Na dimensão psicossocial avaliada pelos pais, houve associação significativa em 2018 com a autopercepção de saúde (p=0,035) e limítrofe com a classificação do SDQ total (dificuldades) (p=0,054), e em 2021 não houve associação significante ao nível de 5% entre esse domínio e as variáveis testadas. Com relação à QV geral avaliada pelos pais, em 2018 esta foi associada à autopercepção de saúde (p=0,021) e à classificação do SDQ total (p=0,027), e em 2021, com as mesmas variáveis, autopercepção de saúde (p=0,043) e classificação do SDQ total (p=0,034).

 Ao considerar as respostas dos adolescentes, em 2018 houve associação significativa da dimensão física com a classificação do SDQ total (p=0,003), e em 2021 com a autopercepção de saúde (p=0,001). Na dimensão psicossocial avaliada pelos adolescentes, houve associação significativa em 2018 com idade (p=0,036), ano escolar (p=0,021) e Meta Aprender (p=0,015), com a classificação do SDQ total (p) e limítrofe com a Meta Performance-evitação (p=0,054). Em 2021, o domínio psicossocial foi associado ao sexo (p=0,048), de forma limítrofe com a autopercepção de saúde (p=0,054) e com a classificação do SDQ total (p=0,009). Com relação à QV geral avaliada pelos adolescentes, em 2018 esta foi associada à idade (p=0,045), ao ano escolar (p=0,017), à Meta Aprender (p=0,028) e à classificação do SDQ total (p). Em 2021, o escore geral da QV foi associado à autopercepção de saúde (p=0,026), à Meta Performance-evitação (p=0,002) e ao SDQ total (p=0,012) (Tabelas 1 e 2 ). 

 Para a análise multivariada, foram construídos quatro modelos. No primeiro modelo, avaliou-se a QV pela percepção dos pais em 2018 e as variáveis ano escolar e SDQ total, sendo a última associada à QV no modelo final. Assim, o resultado normal do SDQ aumentou em 5,37 vezes as chances de o adolescente ter boa/excelente qualidade de vida na percepção dos pais. No segundo modelo, avaliou-se a QV pela percepção dos adolescentes em 2018 e as variáveis ano escolar, Meta Aprender e Meta Performance-evitação. No modelo final, a QV foi associada ao ano escolar, sendo que estar matriculado no 6º e no 7º ano aumentou em 4,5 e 9,7 vezes, respectivamente, as chances de ter melhor QV quando comparados aos alunos do 9º ano. No terceiro modelo, avaliou-se a QV segundo a percepção dos pais em 2021. As variáveis autopercepção de saúde e SDQ total foram selecionadas, sendo a última associada à QV no modelo final (ajustado por idade). O resultado normal do SDQ aumentou em 5,51 vezes as chances de o adolescente ter boa/excelente qualidade de vida na percepção dos pais. No quarto modelo, avaliou-se a QV segundo a percepção dos adolescentes em 2021, sendo selecionadas para entrar no modelo inicial as variáveis sexo, CCEB, autopercepção de saúde, Meta Performance-evitação e SDQ total. Essas duas últimas variáveis permaneceram associadas à QV no modelo final, de forma que ter melhor resultado na escala de performance-evitação (baixa evitação da aprendizagem) aumentou em 5,02 vezes as chances de ter melhor QV, e o resultado normal do SDQ aumentou em 11,05 vezes as chances de o adolescente ter melhor QV ( Tabela 3 ). 

**Tabela 1:** - Análise bivariada da associação entre as variáveis do PedsQL ^*^ (pais e adolescentes) e variáveis selecionadas (n = 68). Belo Horizonte, MG, Brasil, 2018

**Características**	**Pais** **Dimensões**						**Adolescentes** **Dimensões**					
	**Física**		**Psicossocial**		**Qualidade de vida geral**		**Física**		**Psicossocial**		**Qualidade de vida geral**	
	alta	baixa	alta	baixa	alta	baixa	alta	baixa	alta	baixa	alta	baixa
	n(%) ^†^	n(%) ^†^	n(%) ^†^	n(%) ^†^	n(%) ^†^	n(%) ^†^	n(%) ^†^	n(%) ^†^	n(%) ^†^	n(%) ^†^	n(%) ^†^	n(%) ^†^
**Sexo**												
Feminino	19 (55,9)	20 (58,8)	21 (52,5)	18 (64,3)	19 (51,4)	20 (64,5)	24 (66,7)	15 (46,9)	18 (54,5)	21 (60,0)	17 (53,1)	22 (61,1)
Masculino	15 (44,1)	14 (41,2)	19 (47,5)	10 (35,7)	18 (48,6)	11 (35,5)	12 (33,3)	17 (53,1)	15 (45,5)	14 (40,0)	15 (46,9)	14 (38,9)
Valor – ^p‡^	0,806 ^§^		0,333 ^§^		0,274 ^§^		0,100 ^§^		0,649 ^§^		0,506 ^§^	
**Idade (anos)**												
11	7 (20,6)	11 (32,4)	10 (25,0)	8 (28,6)	8 (21,6)	10 (32,3)	11 (30,6)	7 (21,9)	7 (21,2)	11 (31,4)	7 (21,9)	11 (30,6)
12	6 (17,6)	8 (23,5)	8 (20,0)	6 (21,4)	8 (21,6)	6 (19,4)	6 (16,7)	8 (25,0)	5 (15,2)	9 (25,7)	5 (15,6)	9 (25,0)
13	8 (23,5)	9 (26,5)	9 (22,5)	8 (28,6)	9 (24,3)	8 (25,8)	7 (19,4)	10 (31,3)	7 (21,2)	10 (28,6)	6 (18,8)	11 (30,6)
14	13 (38,2)	6 (17,6)	13 (32,5)	6 (21,4)	12 (32,5)	7 (22,6)	12 (33,3)	7 (21,9)	14 (42,4)	5 (14,3)	14 (43,8)	5 (13,9)
Valor – p ^‡^	0,077 ^||^		0,494 ^||^		0,309 ^||^		0,932 ^||^		**0,036** ^	|^	**0,045** ^|	^
**Ano escolar**												
6º	7 (20,6)	13 (38,2)	10 (25,0)	10 (35,7)	8 (21,6)	12 (38,7)	12 (33,3)	8 (25,0)	8 (24,2)	12 (34,3)	8 (25,0)	12 (33,3)
7º	7 (20,6)	10 (29,4)	10 (25,0)	7 (25,0)	9 (24,3)	8 (25,8)	5 (13,9)	12 (37,4)	5 (15,2)	12 (34,3)	4 (12,5)	13 (36,1)
8º	9 (26,5)	6 (17,6)	10 (25,0)	5 (17,9)	11 (29,7)	4 (12,9)	9 (25,0)	6 (18,8)	8 (24,2)	7 (20,0)	8 (25,0)	7 (19,4)
9º	11 (32,4)	5 (14,7)	10 (25,0)	6 (21,4)	9 (24,3)	7 (22,6)	10 (27,8)	6 (18,8)	12 (36,4)	4 (11,4)	12 (37,5)	4 (11,1)
Valor – p ^‡^	**0,027** ^	|^	0,377 ^||^		0,181 ^||^		0,567 ^||^		**0,021** ^	|^	**0,017** ^|	^
**CCEB** ^¶^												
A1 ^**^	21 (61,8)	19 (55,9)	23 (57,5)	17 (60,7)	21 (56,8)	19 (61,3)	22 (61,1)	18 (56,3)	21 (63,6)	19 (54,3)	20 (62,5)	20 (55,6)
B1/B2 ^††^	13 (38,2)	15 (44,1)	17 (42,5)	11 (39,3)	16 (43,2)	12 (38,7)	14 (38,9)	14 (43,8)	12 (36,4)	16 (45,7)	12 (37,5)	16 (44,4)
Valor – p ^‡^	0,622		0,791 ^§^		0,705 ^§^		0,684 ^§^		0,434 ^§^		0,561 ^§^	
**Autopercepção de saúde**												
Ruim/Regular	5 (14,7)	1 (2,9)	6 (15,0)	0 (0,0)	6 (16,2)	0 (0,0)	4 (11,1)	2 (6,3)	4 (12,1)	2 (5,7)	4 (12,5)	2 (5,6)
Boa/Excelente	29 (85,3)	33 (97,1)	34 (85,0)	28 (100,0)	31 (83,8)	31 (100,0)	32 (88,9)	30 (93,7)	29 (87,9)	33 (94,3)	28 (87,5)	34 (94,4)
Valor – p ^‡^	0,099 ^‡‡^		**0,035** ^‡‡^		**0,021** ^‡‡^		0,395 ^‡‡^		0,308 ^‡‡^		0,281 ^‡‡^	
**Meta Aprender**												
< 31	18 (52,9)	17 (50,0)	21 (52,5)	14 (50,0)	20 (54,1)	15 (48,4)	20 (55,6)	15 (46,9)	22 (66,7)	13 (37,1)	21 (65,6)	14 (38,9)
≥ 31	16 (47,1)	17 (50,0)	19 (47,5)	14 (50,0)	17(45,9)	16 (51,6)	16 (44,4)	17 (53,1)	11 (33,3)	22 (66,9)	11 (34,4)	22 (61,1)
Valor – p ^‡^	0,808 ^§^		0,839 ^§^		0,641 ^§^		0,475 ^§^		**0,015** ^§^		**0,028** ^§^	
**Meta Performance-aproximação**												
< 15	18 (52,9)	15 (44,1)	20 (50,0)	13 (46,4)	19 (51,4)	14 (45,2)	17 (47,2)	16 (50,0)	15 (45,5)	18 (51,4)	14 (43,7)	19 (52,8)
≥ 15	16 (47,1)	19 (55,9)	20 (50,0)	15 (53,6)	18 (48,6)	17 (54,8)	19 (52,8)	16 (50,0)	18 (54,5)	17 (48,6)	18 (56,3)	17 (47,2)
Valor – p ^‡^	0,467 ^§^		0,772 ^§^		0,611 ^§^		0,819 ^§^		0,622 ^§^		0,457 ^§^	
**Meta Performance-evitação**												
≤ 8	19 (55,9)	18 (52,9)	19 (47,5)	18 (64,3)	19 (51,4)	18 (58,1)	19 (52,8)	18 (56,3)	14 (42,4)	23 (65,7)	14 (43,7)	23 (63,9)
> 8	15 (44,1)	16 (47,1)	21 (52,5)	10 (35,7)	18 (48,6)	13 (41,9)	17 (47,2)	14 (43,8)	19 (57,6)	12 (34,3)	18 (56,3)	13 (36,1)
Valor – p ^‡^	0,808 ^§^		0,171 ^§^		0,580 ^§^		0,774 ^§^		**0,054** ^§^		0,096 ^§^	
**SDQ** ^§§^ **Comportamento Pró-social**												
Normal	33 (97,1)	32 (94,1)	39 (97,5)	26 (92,9)	36 (97,3)	29 (93,5)	34 (94,4)	31 (96,9)	31 (93,9)	34 (97,1)	30 (93,8)	35 (97,2)
Alterado ^||^	1 (2,9)	2 (5,9)	1 (2,5)	2 (7,1)	1 (2,7)	2 (6,5)	2 (5,6)	1 (3,1)	2 (6,1)	1 (2,9)	2 (6,3)	1 (2,8)
Valor – p ^‡^	0,500 ^‡‡^		0,367 ^‡‡^		0,433 ^‡‡^		0,545 ^‡‡^		0,478 ^‡‡^		0,455 ^‡‡^	
**SDQ** ^§§^ **Total**												
Normal	25 (73,5)	31 (91,2)	30 (75,0)	26 (92,9)	27 (73,0)	29 (93,5)	25 (69,4)	31 (96,9)	21 (63,6)	35 (100,0)	20 (62,5)	36 (100,0)
Alterado ^||^	9 (26,5)	3 (8,8)	10 (25,0)	2 (7,1)	10 (27,0)	2 (6,5)	11 (30,6)	1 (3,1)	12 (36,4)	0 (0,0)	12 (37,5)	0 (0,0)
Valor – p ^‡^	**0,056** ^§^		**0,054** ^‡‡^		**0,027** ^§^		**0,003** ^§^		**<0,001** ^§^		**<0,001** ^§^	

^*^ PedsQL = Questionário Pediátrico sobre Qualidade de Vida;

^†^ N(%) = Número de participantes e porcentagem de participantes;

^¶^ CCEB = Critério de Classificação Econômica Brasil;

^**^ A1 = Classe A1;

^††^ B1/B2 = Classe B1/B2;

^§§^ SDQ = *The Strengths and Difficulties Questionnaire*

^||^ Associação linear por linear;

^‡^ Valor – p;

^§^ Qui-quadrado de Pearson;

^‡‡^ Exato de Fisher;

**Tabela 2:** - Análise bivariada da associação entre as variáveis do PedsQL ^*^ (pais e adolescentes) e variáveis selecionadas (n = 68). Belo Horizonte, MG, Brasil, 2021

**Características**	**Pais** **Dimensões**						**Adolescentes** **Dimensões**					
	**Física**		**Psicossocial**		**Qualidade de vida geral**		**Física**		**Psicossocial**		**Qualidade de vida geral**	
	alta	baixa	alta	baixa	alta	baixa	alta	baixa	alta	baixa	alta	baixa
	n(%) ^†^	n(%) ^†^	n(%) ^†^	n(%) ^†^	n(%) ^†^	n(%) ^†^	n(%) ^†^	n(%) ^†^	n(%) ^†^	n(%) ^†^	n(%) ^†^	n(%) ^†^
**Sexo**												
Feminino	30 (65,2)	9 (40,9)	18 (62,1)	21 (53,8)	19 (63,3)	20 (52,6)	12 (75,0)	27 (51,9)	28 (66,7)	11 (42,3)	23 (69,7)	16 (47,1)
Masculino	16 (34,8)	13 (59,1)	11 (37,9)	18 (46,2)	11 (36,7)	18 (47,4)	4 (25,0)	25 (48,1)	14 (33,3)	15 (57,7)	10 (30,3)	18 (52,9)
Valor – p ^‡^	0,058 ^§^		0,498 ^§^		0,376 ^§^		0,103 ^§^		0,048 ^§^		0,060 ^§^	
**Idade (anos)**												
11	14 (30,4)	6 (27,3)	9 (31,0)	11 (28,2)	9 (30,0)	11 (28,9)	5 (31,3)	15 (28,8)	13 (31,0)	7 (26,9)	12 (36,4)	8 (23,5)
12	9 (19,6)	7 (31,8)	5 (17,2)	11 (28,2)	6 (20,0)	10 (26,3)	2 (12,5)	14 (26,9)	9 (21,4)	7 (26,9)	5 (15,2)	10 (29,5)
13	13 (28,3)	4 (18,2)	6 (20,7)	11 (28,2)	7 (23,3)	10 (26,3)	5 (31,3)	12 (23,1)	11 (26,2)	6 (23,1)	9 (27,3)	8 (23,5)
14	10 (21,7)	5 (22,7)	9 (31,0)	6 (15,4)	8 (26,7)	7 (18,5)	4 (25,0)	11 (21,2)	9 (21,4)	6 (23,1)	7 (21,2)	8 (23,5)
Valor – p ^‡^	0,867 ^||^		0,451 ^||^		0,653 ^||^		0,678 ^||^		0,882 ^||^		0,623 ^||^	
**Ano escolar**												
8º	1 (2,2)	0 (0,0)	0 (0,0)	1 (2,6)	1 (3,3)	10 (26,3)	1 (6,3)	0 (0,0)	1 (2,4)	0 (0,0)	1 (3,0)	0 (0,0)
9º	13 (28,3)	8 (36,4)	9 (31,0)	12 (30,8)	9 (30,0)	12 (31,6)	5 (31,3)	16 (30,8)	12 (28,6)	9 (34,6)	11 (33,3)	10 (29,4)
1º ano EM ^¶^	11 (23,9)	5 (22,7)	6 (20,7)	10 (25,6)	6 (20,0)	10 (26,3)	1 (6,3)	15 (28,8)	10 (23,8)	6 (23,1)	6 (18,2)	9 (26,5)
2º ano EM ^¶^	10 (21,7)	4 (18,2)	5 (17,3)	9 (23,1)	5 (16,7)	9 (23,9)	3 (18,8)	11 (21,2)	8 (19,0)	6 (23,1)	6 (18,2)	8 (23,5)
3º ano EM ^¶^	11 (23,9)	5 (22,7)	9 (31,0)	7 (17,9)	9 (30,0)	7 (18,4)	6 (37,5)	10 (19,2)	11 (26,2)	5 (19,2)	9 (27,3)	7 (20,6)
Valor – p ^‡^	0,754 ^||^		0,389 ^||^		0,704 ^||^		−		0,707 ^||^		0,947 ^||^	
**CCEB** ^**^												
A1 ^††^	28 (60,9)	12 (54,5)	17 (58,6)	23 (59,0)	19 (63,3)	21 (55,3)	12 (75,0)	28 (53,8)	27 (64,3)	13 (50,0)	22 (66,7)	17 (50,0)
B1/B2 ^‡‡^	18 (39,1)	10 (45,5)	12 (41,4)	16 (41,0)	11 (36,7)	17 (44,7)	4 (25,0)	24 (46,2)	15 (35,7)	13 (50,0)	11 (33,3)	17 (50,0)
Valor – p ^‡^	0,620 ^§^		0,977 ^§^		0,502 ^§^		0,133		0,245 ^§^		0,167 ^§^	
**Autopercepção de saúde**												
Ruim/Regular	12 (26,1)	1 (4,5)	7 (24,1)	6 (15,4)	9 (30,0)	4 (10,5)	8 (50,0)	5 (9,6)	11 (26,2)	2 (7,7)	10 (30,3)	3 (8,8)
Boa/Excelente	34 (73,9)	21 (95,5)	22 (75,9)	33 (84,6)	21 (70,0)	34 (89,5)	8 (50,0)	47 (90,4)	31 (73,8)	24 (92,3)	23 (69,7)	31 (91,2)
Valor – p ^‡^	0,031 ^§§^		0,364 ^§§^		0,043 ^§§^		0,001 ^§§^		0,054 ^§§^		0,026 ^§^	
**Meta Aprender**												
< 31	30 (65,2)	13 (59,1)	20 (69,0)	14 (50,0)	20 (66,7)	23 (60,50)	10 (62,5)	33 (63,5)	28 (66,7)	15 (57,7)	22 (66,7)	20 (58,8)
≥ 31	16 (34,8)	9 (40,9)	9 (31,0)	14 (50,0)	10 (33,3)	15 (39,5)	6 (37,5)	19 (36,5)	14 (33,3)	11 (42,3)	11 (33,3)	14 (41,2)
Valor – p ^‡^	0,624 ^§^		0,398 ^§^		0,602 ^§^		0,944 ^§^		0,456 ^§^		0,507 ^§^	
**Meta Performance-aproximação**												
< 15	21 (45,7)	18 (81,8)	17 (58,6)	22 (56,4)	17 (56,7)	22 (57,9)	10 (62,5)	29 (55,8)	23 (54,8)	16 (61,5)	19 (57,6)	20 (58,8)
≥ 15	25 (54,3)	4 (18,2)	12 (41,4)	17 (43,6)	13 (43,3)	16 (42,1)	6 (37,5)	23 (44,2)	19 (45,2)	10 (38,5)	14 (42,4)	14 (47,2)
Valor – p ^‡^	0,005 ^§^		0,855 ^§^		0,919 ^§^		0,634 ^§^		0,583 ^§^		0,918 ^§^	
**Meta Performance-evitação**												
≤ 8	22 (47,8)	10 (45,5)	14 (48,3)	18 (46,2)	14 (46,7)	18 (47,4)	4 (25,0)	28 (53,8)	16 (38,1)	16 (61,5)	9 (27,3)	22 (64,7)
> 8	24 (52,2)	12 (54,5)	15 (51,7)	21 (53,8)	16 (53,3)	20 (52,6)	12 (75,0)	24 (46,2)	26 (61,9)	10 (38,5)	24 (72,7)	12 (35,3)
Valor – p ^‡^	0,855 ^§^		0,862 ^§^		0,954 ^§^		0,043 ^§^		0,060 ^§^		0,002 ^§^	
**SDQ** ^||||^ **Comportamento Pró-social**												
Normal	46 (100,0)	21 (95,5)	29 (100,0)	38 (97,4)	30 (100,0)	37 (97,4)	16 (100,0)	51 (98,1)	41 (97,6)	26 (100,0)	32 (97,0)	34 (100,0)
Alterado ^||^	0 (0,0)	1 (4,5)	0 (0,0)	1 (2,6)	0 (0,0)	1 (2,6)	0 (0,0)	1 (1,9)	1 (2,4)	0 (0,0)	1 (3,0)	0 (0,0)
Valor – p ^‡^	0,324 ^§§^		0,574 ^§^		0,559 ^§§^		0,765 ^§§^		0,618 ^§§^		0,493 ^§§^	
**SDQ* ^||||^ **Total**												
Normal	40 (87,0)	19 (86,4)	23 (79,3)	36 (92,3)	23 (76,7)	36 (94,7)	12 (75,0)	47 (90,4)	33 (78,6)	26 (100,0)	25 (75,8)	33 (97,1)
Alterado ^||^	6 (13,0)	3 (13,6)	6 (20,7)	3 (7,7)	7 (23,3)	2 (5,3)	4 (25,0)	5 (9,6)	9 (21,4)	0 (0,0)	8 (24,2)	1 (0,0)
Valor – p ^‡^	0,610 ^§§^		0,115 ^§§^		0,034 ^§§^		0,124 ^§§^		0,009 ^§§^		0,012 ^§§^	

^*^ PedsQL = Questionário Pediátrico sobre Qualidade de Vida;

^†^ N(%) = Número de participantes e porcentagem de participantes ;

^¶^ EM = Ensino Médio;

^**^ CCEB = Critério de Classificação Econômica Brasil;

^††^ A1 = Classe A1;

^‡‡^ B1/B2 = Classe B1/B2;

^§^ Qui-quadrado de Pearson;

^||||^ SDQ = *The Strengths and Difficulties Questionnaire*

^||^ Associação linear por linear;

^‡^ Valor – p;

^§§^ Exato de Fisher;

**Tabela 3:** - Resultados das análises de regressão logística multivariada entre qualidade de vida (PedsQL ^*^ ) segundo pais e adolescentes (n = 68). Belo Horizonte, MG, Brasil, 2018 e 2021

**Características**		**Modelo 1** ^†^ **- Qualidade de Vida Geral Pais 2018**				**Modelo 2** ^‡^ **- Qualidade de Vida Geral Adolescentes 2018**			
		Modelo inicial		Modelo final		Modelo inicial		Modelo final	
		OR (IC95%) ^§^	Valor-p ^||^	OR (IC95%) ^§^	Valor-p ^||^	OR (IC 95%) ^§^	Valor-p ^¶^	OR (IC 95%) ^§^	Valor-p ^¶^
Ano Escolar	6º	1,92 (0,47-7,90)	0,368	−	−	4,31 (0,99-18,78)	0,052	**4,50 (1,06-19,04)**	**0,041**
	7º	0,85 (0,20-3,58)	0,826	−	−	11,06 (2,15-56,72)	0,004	**9,75 (1,98-47,94)**	**0,005**
	8º	0,41 (0,08-1,97)	0,264	−	−	2,28 (0,48-10,76)	0,299	2,65 (0,57-11,99)	0,213
Autopercepção de saúde		−	−	−	−	−	−	−	−
Meta Aprender		−	−	−	−	2,07 (0,66-6,53)	0,212	−	−
Meta Performance-Evitação		−	−	−	−	1,61 (0,54-4,82)	0,392		
SDQ ^**^ Comportamento Pró-Social		−	−	−	−	−	−	−	−
SDQ ^**^ (total)		6,21 (1,16-33,16)	0,033	**5,35 (1,06-26,9)**	**0,042**	−	−	−	−
**Características**		**Modelo 3** ^††^ **- Qualidade de Vida Geral Pais 2021**				**Modelo 4** ^‡‡^ **- Qualidade de Vida Geral Adolescentes 2021**			
		Modelo inicial		Modelo final		Modelo inicial		Modelo final	
		OR (IC 95%) ^§^	Valor-p ^§§^	OR (IC 95%) ^§^	Valor-p ^§§^	OR (IC 95%) ^§^	Valor-p ^||||^	OR (IC 95%) ^§^	Valor-p ^||||^
Sexo		−	−	−	−	1,78 (0,56-5,62)	0,325		
CCEB ^¶¶^						2,26 (0,71-7,19)	0,167	−	−
Autopercepção de saúde		2,93 (0,76-11,29)	0,118	−	−	2,52 (0,51-12,38)	0,256	−	−
Meta Performance-Evitação		−	−	−	−	4,44 (1,39-14,11)	0,011	**5,02 (1,67-15,12)**	**0,004**
SDQ ^**^ Comportamento Pró-Social		−	−	−	−	−	−	−	−
SDQ ^**^ (total)		4,36 (0,79-23,96)	0,090	**5,51 (1,05-28,98)**	**0,044**	7,83 (0,78-78,58)	0,080	**11,05 (1,18-103,77)**	**0,035**

^*^ PedsQL = Questionário Pediátrico sobre Qualidade de Vida;

^†^ Modelo 1 = 9º ano, SDQ Total: Alterado, Autopercepção de saúde ruim/regular não entrou no modelo pois não convergiu devido à categoria sem observações, Modelo Final ajustado por idade;

^‡^ Modelo 2 = 9º ano, Autopercepção de saúde ruim/regular, Meta Aprender e Meta Performance-evitação: maior escore, SDQ não entrou no modelo pois não convergiu devido à categoria sem observações;

^||^ Teste de Wald, Ajuste dos modelos inicial/final (Hosmer e Lemeshow): p=0,638/p=0,576;

^¶^ Teste de Wald, Ajuste dos modelos inicial/final (Hosmer e Lemeshow): p=0,639/p=1,000;

^††^ Modelo 3 = Autopercepção de saúde ruim/regular, SDQ Total: Alterado, Modelo Final ajustado por idade;

^‡‡^ Modelo 4 = Sexo masculino, Autopercepção de saúde ruim/regular, Meta Performance-evitação: maior escore, SDQ Total: Alterado;

^§^ OR(IC95%) = *Odds Ratio* e intervalo de confiança de 95%;

^§§^ Teste de Wald, Ajuste dos modelos inicial/final (Hosmer e Lemeshow): p=0,713/p=0,834;

^||||^ Teste de Wald, Ajuste dos modelos inicial/final (Hosmer e Lemeshow): p=0,700/p=0,289;

^¶¶^ CCEB = Critério de Classificação Econômica Brasil

^**^ SDQ = *The Strengths and Difficulties Questionnaire* ;

Em síntese, considerando as análises de regressão logística nos dois períodos, a variável Capacidades e Dificuldades, medida pelo escore total do SDQ, testada nos modelos 1 e 3, foi associada à qualidade de vida na avaliação dos pais em 2018 e 2021. Segundo a percepção dos adolescentes, a qualidade de vida foi associada, em 2018, ao ano escolar e em 2021, à Meta Performance-evitação e ao SDQ total.

## Discussão

 A saúde e os determinantes sociais da saúde encontram, no ambiente da escola, campo de estudo e reflexões, por ser a idade escolar um período crucial de crescimento físico e cognitivo ^(^ ^27^ ^)^ . Por ser o contexto da pesquisa uma instituição de financiamento privado, os participantes pertenciam a estratos mais altos no que se refere à classe econômica. Na comparação entre os períodos 2018 e 2021, houve piora da avaliação de autopercepção de saúde, já que 8,8% e 19,2%, respectivamente, consideraram a sua saúde como regular, ruim ou muito ruim, pesando a situação de emergência em saúde pública. A suspensão das aulas presenciais e a adaptação da rotina e dos ciclos escolares ao contexto remoto causaram insegurança e incertezas, e o isolamento social, com a diminuição do contato com pares, afetou o bem-estar dos adolescentes ^(^ ^17^ ^-^ ^18^ ^)^ . A escola é um espaço coletivo favorável à promoção da saúde, que oferece às crianças e adolescentes um campo de conscientização e prática de hábitos de vida saudáveis, autocuidado e prevenção de riscos ^(4,15).^

 Sendo a adolescência uma fase crítica e sensível da vida do indivíduo e suas famílias, quando são experimentadas profundas transformações físicas, emocionais ^(^ ^28^ ^)^ , sociais e cognitivas, é importante monitorar a saúde e o bem-estar de diferentes aspectos, sendo a qualidade de vida um indicador bastante apropriado ^(^ ^29^ ^)^ . Sobre a avaliação da qualidade de vida na percepção dos pais, o aumento do escore da dimensão psicossocial (emocional, social e escolar) evidenciado pelo estudo pode ser explicado pelo uso dos recursos tecnológicos acessíveis ao estrato social estudado, e a diminuição do escore dimensão física reflete a crise sanitária e epidemiológica ocorrida em decorrência da disseminação do SARS-CoV-2. Além do prejuízo na esfera educacional, o fechamento das escolas gerou uma mudança significativa na dinâmica das famílias, que precisaram compatibilizar os compromissos profissionais dos pais com as demandas estudantis dos adolescentes em maiores espaços de tempo compartilhados ^(^ ^30^ ^)^ , o que envolveu maior interação dos pais na vida escolar dos adolescentes ^(^ ^14^ ^)^ . Quando observada a qualidade de vida a partir do autorrelato dos adolescentes, a diminuição dos escores na dimensão psicossocial pode ser justificada pela alta demanda de interação com pares inerente à fase da adolescência, que foi prejudicada pelas necessárias medidas de distanciamento social. A interação e a comunicação são, sabidamente, necessidades naturais dos seres humanos ^(^ ^10^ ^)^ e o ambiente escolar é lugar de crescimento, desenvolvimento de habilidades cognitivas e comportamentais e socialização com repercussões na qualidade de vida da criança e do adolescente ^(^ ^8^ ^,^ ^31^ ^)^ . 

 Em acréscimo, atrelados à qualidade de vida, aspectos como sexo, escolaridade do pai e da mãe e renda familiar foram investigados em um estudo realizado com adolescentes iranianos com idade entre 15 e 18 anos, que evidenciou efeitos significativos da desigualdade socioeconômica na qualidade de vida relacionada à saúde de adolescentes iranianos, indicando a necessidade de políticas públicas em prol de uma sociedade saudável para melhor qualidade de vida ^(^ ^29^ ^)^ . 

 Na análise da motivação para aprender, na comparação entre os períodos estudados, foi evidenciada queda da Meta Aprender e o aumento da Meta Performance-evitação, condições determinadas, possivelmente, por questões de ordem pedagógica, com a implementação súbita de um modelo remoto de ensino. Adaptar o sistema educacional, envolvendo docentes e discentes em torno de conteúdos, métodos de ensino, de aprendizagem e de avaliação é um dos desafios deste momento histórico, já que a motivação dos estudantes a aprender passa, também, por questões de ordem docente, estrutural e social ^(^ ^32^ ^)^ . No que diz respeito à inserção das tecnologias de informação e comunicação, uma pesquisa com estudantes brasileiros do Ensino Médio deu a conhecer que 20% dos participantes negaram a suficiência das aulas remotas. O principal motivo foi a dificuldade em compreender o conteúdo transmitido, resultado que faz pensar sobre a interação remota entre professor e aluno e o quão motivadora e efetiva ela pode ser para a educação básica ^(^ ^33^ ^)^ . 

 A interrupção da rotina escolar presencial também repercutiu no interesse de adolescentes de uma instituição pública da região sudeste do Brasil - região que não concentra os maiores percentuais de exclusão escolar do país -, onde quase 40% dos estudantes sinalizaram desinteresse nas atividades escolares à distância e outros 49% manifestaram propensão à evasão escolar ^(^ ^2^ ^)^ . Com relação ao segmento dos anos finais do Ensino Fundamental, os resultados de outra pesquisa realizada no período pandêmico mostraram que os estudantes habitualmente motivados intrinsecamente para aprender, apresentaram-se motivados extrinsecamente ou desmotivados no que se refere ao ensino remoto, sugerindo implicações diretas entre a qualidade do envolvimento entre professor e aluno e a motivação para aprender nesta modalidade de ensino ^(^ ^34^ ^)^ . Para os estudantes universitários, a retomada das atividades presenciais representou melhora dos níveis motivacionais para o estudo, permeado pelo contato social ^(^ ^35^ ^)^ . 

 Nesse sentido, os valores de razões de chances encontrados nas análises de regressão logística multivariada realizadas com a amostra do presente estudo mostraram resultados interessantes. As maiores chances de ter melhor qualidade de vida autorreferida encontradas entre os adolescentes dos anos iniciais do Ensino Fundamental II corroboraram estudos pré-pandêmicos ^(^ ^36^ ^-^ ^37^ ^)^ , que mostraram que estudantes mais novos apresentam melhor qualidade de vida geral quando comparados aos estudantes dos anos finais em diferentes estratos educacionais. Uma investigação longitudinal realizada em 2018 com estudantes adolescentes chineses evidenciou queda da satisfação com a vida e aumento gradual da desesperança no percurso acadêmico, associados a aspectos individuais e contextuais, como resiliência, fatores socioculturais e ambiente familiar ^(^ ^38^ ^)^ . 

 Em ambos os períodos investigados, o comportamento típico do adolescente favoreceu que ele tivesse boa ou excelente qualidade de vida na percepção dos seus pais e em sua própria avaliação, onde ter o resultado normal na análise das capacidades e dificuldades, relacionado à saúde mental do estudante, aumentou em mais de cinco vezes as chances de ter melhor qualidade de vida. Este dado sugere a aproximação entre os construtos de saúde mental e qualidade de vida. A atuação conjunta de profissionais da saúde e da educação na escola contribui para a implementação e desenvolvimento de ações para o adolescente em busca do comportamento saudável frente a si mesmo, de suas atividades e do grupo do qual faz parte ^(^ ^3^ ^,^ ^39^ ^-^ ^40^ ^)^ . 

 Assim, também é importante destacar o vínculo existente entre a saúde física e psicossocial e o comportamento motivacional adotado pelo adolescente frente à aprendizagem, já que estudantes que apresentaram resultado adequado na análise das capacidades e dificuldades e melhor qualidade de motivação para aprender, indicada pela baixa evitação da aprendizagem, tiveram chances entre cinco e onze vezes maiores de ter melhor qualidade de vida geral na análise do período pandêmico. A saúde, em suas múltiplas dimensões, dialoga com a conduta adequada no ambiente escolar, incluindo o cumprimento das tarefas acadêmicas. A presença de fatores de risco para problemas de saúde mental, por exemplo, afetou a qualidade de vida relacionada à saúde de adolescentes alemães, como mostrou um estudo longitudinal em 2017 ^(^ ^41^ ^)^ , estando em consonância com o impacto negativo na qualidade de vida observado em jovens universitários brasileiros com sintomas de depressão, ansiedade e estresse ^(^ ^42^ ^)^ . 

 Condições psicológicas e distúrbios emocionais não são raros entre adolescentes ^(^ ^43^ ^)^ , e a pandemia causada pelo SARS-CoV-2 contribuiu com a epidemiologia destes transtornos. Segundo estudos chineses realizados em 2020, com adolescentes de 12 a 18 anos e jovens, é alta a prevalência de sintomas depressivos, de ansiedade, estresse pós-traumático e outros problemas psicológicos. O acesso às informações sobre a doença e medidas de prevenção foi considerado fator de proteção contra o desenvolvimento dos sintomas emocionais ^(^ ^19^ ^-^ ^20^ ^)^ . Da mesma forma, pesquisas conduzidas com crianças e adolescentes na Alemanha em 2020 ^(^ ^21^ ^)^ e no Brasil em 2022 ^(^ ^18^ ^)^ indicaram que dois terços dos participantes relataram estar altamente sobrecarregados e que os adolescentes manifestaram o aumento do sentimento de solidão, ansiedade e tristeza em função da pandemia. A qualidade de vida relacionada à saúde autorreferida foi significativamente menor quando comparada ao período anterior à crise sanitária, além de relatarem mais problemas de saúde mental e níveis de ansiedade mais elevados. Ademais, crianças e adolescentes com nível socioeconômico mais baixo foram mais impactados, reforçando a necessidade da implementação de estratégias de promoção de saúde e prevenção de doenças para manter a saúde mental e melhorar a qualidade de vida das crianças e adolescentes, especialmente para as que estão em situação de maior vulnerabilidade ^(^ ^44^ ^)^ . 

O presente estudo sinalizou repercussões de caráter pedagógico, formativo, de saúde física e socioemocional de adolescentes que cursavam os anos finais do Ensino Fundamental no período da pandemia causada pelo SARS-CoV-2. A perda de seguimento de uma parte do grupo pesquisado em 2018 constituiu-se em uma limitação da pesquisa. Por se tratar de uma amostra de um único estrato social, estudantes de uma instituição de financiamento privado, os resultados não representam a massiva realidade brasileira, sendo desejáveis outras pesquisas para ampliar as conclusões.

As contribuições do presente trabalho para o avanço do conhecimento científico na área da saúde perpassam uma gama de fatores, intrínsecos e extrínsecos ao indivíduo, inerentes ao desenvolvimento da infância e adolescência, em que a atuação intersetorial entre os serviços de saúde e instituições educacionais pode favorecer a detecção precoce de agravos, promovendo a saúde em seu sentido ampliado e se constituindo em um campo significativo de atuação para os profissionais da Enfermagem.

## Conclusão

A maioria dos participantes era do sexo feminino e pertencia a classe A. Na comparação entre 2018 e 2021 houve piora da avaliação de autopercepção de saúde. A média das respostas sobre o valor atribuído à saúde ficou estável nos dois períodos. Na avaliação da QV pelos pais, houve diferença estatística significativa entre os escores das dimensões social e psicossocial, ambas com maiores escores em 2021. Entre os adolescentes, houve diferenças entre os escores da QV nas dimensões física, social, escolar, emocional e psicossocial. Para as capacidades e dificuldades não houve diferença significativa no percentual de respostas entre as categorias nas duas etapas do estudo. O adolescente com escore normal na avaliação da saúde mental teve maior chance de ter melhor qualidade de vida nos períodos investigados (OR=5,35 e OR=5,51). Estudantes nos anos iniciais do Ensino Fundamental II apresentaram mais motivação para aprender, sendo que aumentaram em até 9,7 e 5,02 vezes a chance de melhoria da qualidade de vida autorreferida nos dois períodos, respectivamente.

Em síntese, a associação entre saúde, qualidade de vida, comportamento e motivação para aprender de estudantes adolescentes suscita importantes reflexões sobre as abordagens em saúde dos adolescentes no ambiente da escola. Destaca-se a premência de um olhar mais amplo para esse espaço, que deve cumprir importantes funções além das educacionais curriculares, atribuindo significados e construindo uma educação cada vez mais efetiva e abrangente.
